# Functions of the FGF signalling pathway in cephalochordates provide insight into the evolution of the prechordal plate

**DOI:** 10.1242/dev.200252

**Published:** 2022-05-16

**Authors:** Lydvina Meister, Hector Escriva, Stéphanie Bertrand

**Affiliations:** Sorbonne Université, CNRS, Biologie Intégrative des Organismes Marins, BIOM, F-66650 Banyuls-sur-Mer, France

**Keywords:** Amphioxus, Head mesoderm, Notochord, Goosecoid, Brachyury

## Abstract

The fibroblast growth factor (FGF) signalling pathway plays various roles during vertebrate embryogenesis, from mesoderm formation to brain patterning. This diversity of functions relies on the fact that vertebrates possess the largest FGF gene complement among metazoans. In the cephalochordate amphioxus, which belongs to the chordate clade together with vertebrates and tunicates, we have previously shown that the main role of FGF during early development is the control of rostral somite formation. Inhibition of this signalling pathway induces the loss of these structures, resulting in an embryo without anterior segmented mesoderm, as in the vertebrate head. Here, by combining several approaches, we show that the anterior presumptive paraxial mesoderm cells acquire an anterior axial fate when FGF signal is inhibited and that they are later incorporated in the anterior notochord. Our analysis of notochord formation in wild type and in embryos in which FGF signalling is inhibited also reveals that amphioxus anterior notochord presents transient prechordal plate features. Altogether, our results give insight into how changes in FGF functions during chordate evolution might have participated to the emergence of the complex vertebrate head.

## Abstract (precis)

**Summary:** FGF signalling inhibition in cephalochordates induces a loss of anteriormost somites. After FGFR inhibition, the presomitic anterior region cells are incorporated in the anterior notochord which transiently present prechordal plate features.

## INTRODUCTION

During embryonic development, the numerous cells that are generated through successive divisions need to communicate in order to build the correct body plan. Although there is a great diversity in the shape of extant animals, the number of cell-cell communication pathways acting during embryogenesis is limited (Pires-daSilva and Sommer, 2003; Nichols et al., 2006; Richards and Degnan, 2009; Babonis and Martindale, 2017). Among them, the fibroblast growth factor (FGF) signalling pathway, which appeared in metazoans (Rentzsch et al., 2008; Rebscher et al., 2009; Ryan et al., 2013; Bertrand et al., 2014), greatly diversified in the chordate lineage, particularly in vertebrates (Itoh and Ornitz, 2004; Popovici et al., 2005), after the two-whole genome duplications that characterize their evolutionary history (Dehal and Boore, 2005). Therefore, the chordate ancestor possessed eight FGF ligand genes and one FGF receptor (FGFR), while only three ligands were present in the ancestor of bilaterians (Oulion et al., 2012). In vertebrates, there are at least 22 ligand genes and four receptors (FGFR) (Itoh and Ornitz, 2004; Popovici et al., 2005); it remains to be understood how this gene expansion has participated in the acquisition of vertebrate-specific morphological features.

Vertebrates belong to the chordate clade together with tunicates (or urochordates) and cephalochordates (i.e. amphioxus). Even if tunicates are the sister group of vertebrates (Bourlat et al., 2006; Delsuc et al., 2006), they present developmental modalities and genomic features that have diverged significantly (Schubert et al., 2006; Garcia-Fernandez et al., 2007; Lemaire, 2011). On the other hand, cephalochordates have retained chordate characteristics shared with vertebrates, such as the presence of a dorsal organizer (Garcia-Fernandez et al., 2007; Yu et al., 2007; Bertrand and Escriva, 2011; Le Petillon et al., 2017), and have at least one conserved orthologue of almost all the genes that have been predicted in the putative chordate ancestor (Holland et al., 2008). Cephalochordates are characterized by a completely segmented paraxial mesoderm, from the most anterior to the posterior region, and hence do not possess an unsegmented head mesoderm that is found in vertebrates. They also show a notochord that grows anterior to the central nervous system: a feature that was proposed to be a derived character (Chen et al., 1995; Holland et al., 1995). Interestingly, important differences exist in the control of the formation of these mesodermic structures between amphioxus and vertebrates. Thus, although the FGF signal does not control posterior somitogenesis during embryo elongation in amphioxus, which is contrary to vertebrates (Sawada et al., 2001; Dubrulle and Pourquié, 2004; Delfini et al., 2005; Naiche et al., 2011), we have shown that it is required for the development of the rostral somites (Bertrand et al., 2011; Bertrand et al., 2015; Aldea et al., 2019). Indeed, when the unique amphioxus FGFR is inhibited from the blastula stage, the presumptive anterior paraxial mesoderm region fails to express *Pax3*/*7*, *Six1/2*, *MRF1* and most of the genes normally expressed in this embryonic territory during gastrulation, resulting in the specific loss of anterior somites (Bertrand et al., 2011; Aldea et al., 2019). This result has major implications for our understanding of the emergence of the vertebrate head, which is devoid of somites. Indeed, although the segmented character of the vertebrate head mesoderm has been the subject to debate for more than two centuries (von Goethe, 1817; Owen, 1848; Goodrich, 1918), morphological and molecular data in diverse vertebrate species support the absence of segmentation, or at least refute a serial homology between the somites and the head mesoderm (Freund et al., 1996; Bothe and Dietrich, 2006; Kuratani, 2008; Kuratani and Adachi, 2016). Hence, understanding the development of amphioxus anterior somites might shed light on how the complex vertebrate head muscles evolved.

In this work, we aimed to understand the behaviour of the amphioxus anterior paraxial mesoderm domain that does not form somites after FGF signalling pathway inhibition. Using different experimental approaches, we show that cells from this embryonic territory express a specific combination of genes and that they integrate the notochord when the FGF signal is inhibited. Moreover, our fine morphological analysis suggests that the anterior notochord in amphioxus behaves transiently as the prechordal plate of vertebrates, and that only the first somite pair seems to be dependent upon FGF signal for its formation. Finally, our data allow us to propose a refined scenario for the appearance of some features of the complex head of vertebrates.

## RESULTS

### Inhibition of the FGF signalling pathway does not lead to cell apoptosis

**Fig. 1.**
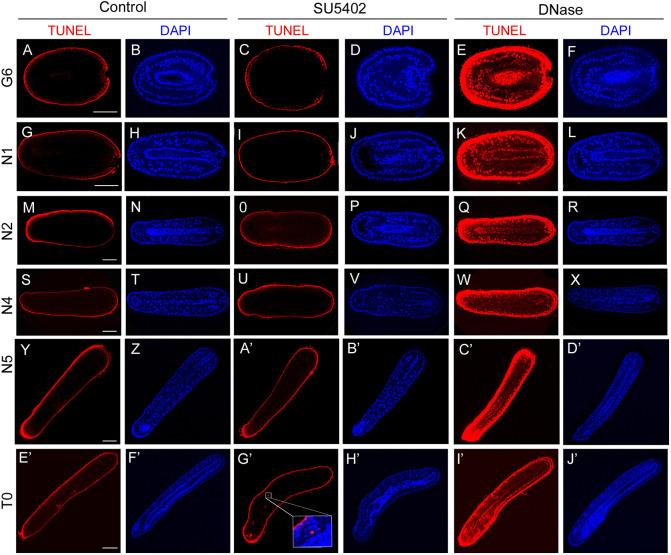
**FGFR inhibition does not induce apoptosis.** TUNEL assay (red) and DAPI (blue) staining in control, SU5402- and DNase I-treated embryos fixed at G6 (A-F), N1 (G-L), N2 (M-R), N4 (S-X), N5 (Y-D′) and T0 (E′-J′) stages. Dorsal views, except for T0 stage embryo pictures (lateral views). Anterior towards the left and dorsal towards the top in T0 stage embryo pictures. The inset shows that the TUNEL labelling does not coincide with the nuclei labelling. At least 10 embryos per stage were analysed and they all showed the same pattern. Scale bars: 50 µm.

Our previous studies showed that a pharmacological treatment of amphioxus embryos at the blastula stage with an inhibitor of FGFR (SU5402) induces a loss of the anterior somites (Bertrand et al., 2011; Aldea et al., 2019). In transverse sections of the anterior region of late neurula stage-treated embryos, no cells can be observed in the region that should correspond to the paraxial mesoderm, and the only visible mesendodermal structures are the notochord dorsally and the endoderm ventrally (Bertrand et al., 2011). However, the fate of the cells of the presumptive anterior paraxial mesoderm that do not form somites in treated embryos was not determined. One hypothesis is that these cells were lost through an apoptotic process. In order to test this proposition, we performed a TUNEL assay in SU5402-treated and control embryos at six developmental stages from late gastrula to transition stage (from stage G6 to T0; Bertrand et al., 2021; Carvalho et al., 2021). In positive control embryos, obtained by using a DNase I treatment, all the nuclei were labelled from the surface to the most internal cells at all stages (Fig. 1). In control embryos, as in SU5402-treated embryos, no labelled nuclei were observed at any stage (Fig. 1). Hence, our results show that the SU5402 treatment did not induce apoptosis of the presumptive anterior paraxial mesoderm cells.

### The presumptive anterior paraxial mesoderm territory expresses the anterior axial mesoderm marker Goosecoid after FGF signalling pathway inhibition

Given that the presumptive anterior paraxial mesoderm cells were not lost by apoptosis after FGF signalling pathway inhibition, we wondered whether the treatment was affecting their fate. We hypothesized that these cells, which are positioned in the mesendoderm epithelial layer between the dorsal axial mesendoderm (which will develop into the future notochord) and the ventral mesendoderm (which later forms the endoderm), acquire the fate of one of these neighbouring territories. In our previous study, we showed that the expression of most of the paraxial dorsal mesendoderm marker genes was abolished in SU5402-treated embryos, with the exception of *Nodal* (Bertrand et al., 2011). We thus decided to undertake double fluorescent *in situ* hybridization (FISH) for *Nodal* and for marker genes of the presumptive endoderm, or for marker genes of the presumptive axial mesoderm.

**Fig. 2.**
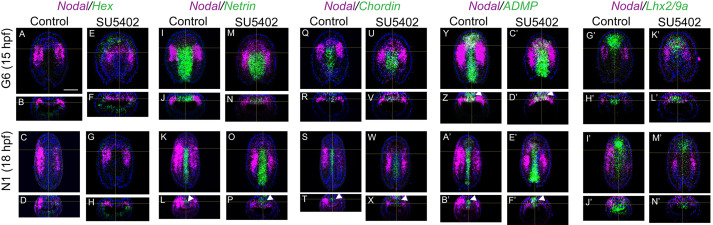
**Double *in situ* hybridization for *Nodal* with *Hex*, *Netrin*, *Chordin*, *ADMP* or *Lhx2/9a*.** Double fluorescent *in situ* hybridization of *Nodal* and the endoderm marker *Hex* (A-H) and of *Nodal* and the dorsal axial markers *Netrin* (I-P), *Chordin* (Q-X), *ADMP* (Y-F′) and *Lhx2/9a* (G′-N′) in control and SU5402-treated embryos fixed at 15 hpf (G6) and 18 hpf (N1). No overlap is observed. Dorsal views and transverse views are shown for *in situ* hybridization data, with anterior towards the top and dorsal towards the top, respectively. Nuclei DAPI staining is in blue. The white arrowheads indicate the neural plate labelling observed for *Netrin*, *Chordin* and *ADMP*. At least five embryos per stage were analysed and they all showed the same pattern. Scale bar: 25 µm.

We analysed gene expression at two developmental times: at 15 hpf, which corresponds to the G6 stage in control embryos, before the first somites form; and at 18 hpf, which corresponds to the N1 stage in controls, when the first three somite pairs are already visible and when *Nodal* expression starts to decrease on the right side (Yu et al., 2002). We observed that the treated embryos were a little bit delayed at 15 hpf, and were hence less elongated than controls (Fig. S1). However, at 18 hpf, although no somites were detected, the SU5402-treated embryos showed an overall size and morphology similar to controls when observed under the light microscope (Fig. S1). In treated embryos, although they had a flatter morphology, the expression of the endodermal gene *Hex* (Yu et al., 2007) was similar to that in control embryos and there was no overlap with *Nodal* expression in either control or treated embryos at 15 hpf or 18 hpf (Fig. 2A-H). Indeed, *Hex* is expressed in both cases in the presumptive endoderm up to the most anterior region, adjacent to the anterior axial *Nodal* expression territory (Fig. S2A-D). We then used *Netrin* (Shimeld, 2000) and *Chordin* (Yu et al., 2007; Somorjai et al., 2008) as presumptive axial mesoderm markers. We observed that in control and SU5402-treated embryos, these genes were expressed in the dorsal axial mesendoderm at 15 hpf, but not in the most anterior region (Fig. 2I,J,M,N,Q,R,U,V) and no overlap with *Nodal* expression was detected. At 18 hpf, *Netrin* expression shifted to the neural plate in both control and treated embryos (Fig. 2K,L,O,P, Fig. S2E,F). At this stage, *Chordin* was expressed in the neural plate of control embryos (Fig. 2S,T, Fig. S2G), whereas in treated embryos both neural plate and underlying axial mesendoderm were labelled (Fig. 2W,X, Fig. S2H), probably due to a little developmental delay compared with controls at this stage. We then analysed the co-expression of *Nodal* with *ADMP* (Yu et al., 2007), *Lhx2/9a*, *Goosecoid* (Neidert et al., 2000) and *Dmbx* (Takahashi and Holland, 2004). At 15 hpf and 18 hpf, *ADMP* was expressed in the dorsal axial mesendoderm from its most anterior region, that is anteriorly continuous with the presumptive endoderm and that does not express *Chordin* or *Netrin* but does express *Nodal*, to the posterior part, in control (Fig. 2Y,Z,A′,B′, Fig. S2I,K) and treated embryos (Fig. 2C′-F′, Fig. S2J,L). *ADMP* was also expressed at the two studied stages in the neural plate region all along the antero-posterior axis of control and SU5402-treated embryos, and no overlap with the paraxial domain region expressing Nodal was observed (Fig. 2Z,B′,D′,F′ and Fig. S2I-L). At 15 hpf and 18 hpf, *Lhx2/9a* expression was observed all along the dorsal axial mesendoderm with an antero-posterior gradient in control and treated embryos, showing no overlap with the *Nodal*-expressing paraxial region (Fig. 2G′-N′).

**Fig. 3.**
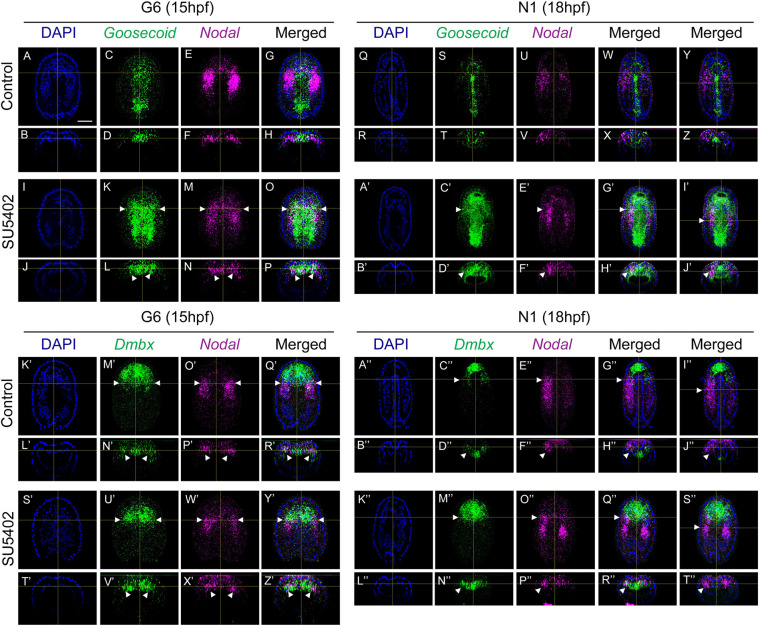
***Nodal* and *Goosecoid* are co-expressed in the anterior dorsal paraxial mesoderm, which also expresses *Dmbx*, after FGFR inhibition.** Double *in situ* hybridization of *Nodal* and *Goosecoid* (A-J′) and of *Nodal* and *Dmbx* (K′-T″) in control and SU5402-treated embryos fixed at 15 hpf (G6) and 18 hpf (N1). Dorsal views and transverse views are shown for *in situ* hybridization data, with anterior towards the top and dorsal towards the top, respectively. Single-channel (DAPI in blue, *Nodal* in magenta, *Goosecoid* and *Dmbx* in green) and merged channel images are shown. For 18 hpf stage embryos, two transverse sections are presented: one at the level of the first somite pair (X,H′,H″,R″) and another one at the level of the second somite pair (Z,J′,J″,T″). The white arrowheads indicate the position of the first somite/presumptive somite pair in panels corresponding to 15 hpf embryos, and the position of the left first (C′-H′,C″-H″,M″-R″) or second somite region (I′,J′,I″,J″,S″,T″) in panels corresponding to 18 hpf embryos. At least five embryos per stage were analysed and they all showed the same pattern. Scale bar: 25 µm.

*Goosecoid* was expressed in control embryos in the axial dorsal mesendoderm and overlying presumptive neural plate at 15 hpf, as previously described (Yu et al., 2007) (Fig. 3A-H, Fig. S2M). At 18 hpf, the expression was observed in the anterior dorsal axial mesendoderm, in the presumptive floor plate in the region posterior to the presumptive cerebral vesicle, and in both the axial mesendoderm and neural plate at the tailbud level (Fig. 3Q-Z, Fig. S2O). In SU5402-treated embryos, the expression was observed at 15 hpf in the whole axial dorsal mesendoderm and in the presumptive neural plate, which are wider than in controls due to the developmental delay. Compared with control embryos, an additional small anterior paraxial domain of expression, overlapping with *Nodal* expression, was detected (Fig. 3I-P, Fig. S2N). At 18 hpf, *Goosecoid* expression was observed in the same axial regions as in controls but was still detected in the dorsal axial mesendoderm in the region posterior to the presumptive cerebral vesicle, again probably due to a little developmental delay (Fig. 3A′-J′, Fig. S2P). However, we could also observe co-expression at this stage with *Nodal* in an anterior paraxial domain corresponding to the first presumptive somite region. Posterior to this domain, no expression overlap in the paraxial mesodermal territory was observed. We also looked at the expression of *Dmbx*. In control and treated embryos, *Dmbx* was expressed at both developmental stages studied in the anteriormost dorsal axial mesendoderm, as well as in the territory of the first presumptive somite pair that also expressed *Nodal* (Fig. 3K′-T″). Altogether, these data show that after inhibition of the FGF signalling pathway, the territory corresponding to the first presumptive somite expresses *Nodal* and *Dmbx* as in wild-type embryos, but that it additionally expresses *Goosecoid*. This combination of gene expression (*Nodal/Dmbx/Goosecoid*) is normally observed only in the anteriormost dorsal axial mesendoderm and it suggests that the first somite territory might acquire an anterior axial fate, although it does not co-express *ADMP* or *Lhx2/9a*.

### The cells of the presumptive anterior paraxial mesoderm are integrated in the anterior notochord after FGF signalling pathway inhibition

**Fig. 4.**
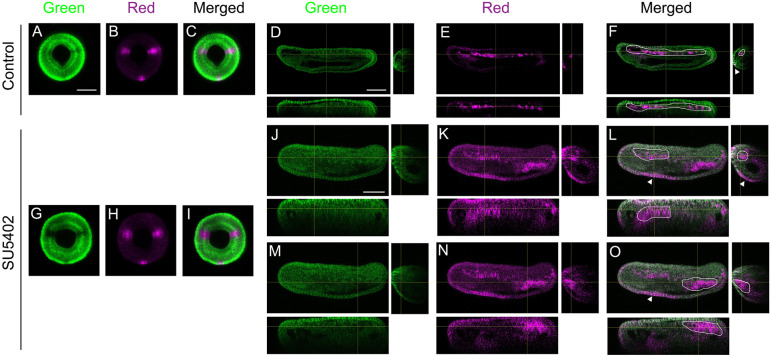
**The presumptive anterior paraxial mesoderm cells are incorporated into the anterior notochord after FGFR inhibition.** One representative embryo is presented for control (*n*=4/4) and SU5402-treated conditions (*n*=7/7). G4 stage control (A-C) and SU5402-treated (G-I) embryos after Kaede protein photoconversion in presumptive paraxial mesoderm and ventral epidermis. Images are blastopore views with dorsal towards the top. The fluorescence was later observed in the corresponding embryos at the T0 stage. (D-F,J-O) Orthogonal views are shown (*xy* below the main panel, *yz* on the right), and single channel as well as merged channel images are presented. For SU5402-treated embryo at T0, two orthogonal views are shown: one at the anterior level (J-L) and one at the posterior level (M-O). In controls (D-F), the red fluorescence (magenta) was observed in the ventral epidermis (arrowhead in F) and in the paraxial mesoderm (encircled by a white line in F) all along the antero-posterior axis. In SU5402-treated embryos (J-O), the red fluorescence was observed in the ventral epidermis (white arrowhead in L and O) as well as in the anterior notochord (encircled by a white line in L) and in the posterior somites (encircled by a white line in O). Scale bars: 50 µm.

To test whether the cells of the presumptive anterior paraxial mesoderm of SU5402-treated embryos expressing the axial mesoderm marker *Goosecoid* are incorporated in the notochord at later stages, we performed a cell-tracing experiment using the Kaede photoconvertible protein (Ando et al., 2002). Following injection of the Kaede mRNA in unfertilized eggs, the embryos were raised in seawater or in seawater with SU5402 added at the blastula stage. When the embryos reached the gastrula stage (G4, 10 hpf), before they start swimming through the beating of cilia, we photoconverted the Kaede protein in the presumptive paraxial mesoderm territory. We also converted a small region of the ectoderm on the opposite side of the embryo (presumptive ventral epidermis) to later test whether we correctly converted Kaede in the target cells (Fig. 4A-C,G-I). The embryos were then immobilized at the late neurula stage (N5, 30 hpf) and imaged alive. In the control embryos that showed red fluorescence in the ventral epidermis, as expected, the somites were labelled red all along the body axis (Fig. 4D-F, *n*=4/4). On the other hand, in the correctly photoconverted treated embryos, although the posterior somites were red, the notochord was labelled in the anterior part of the embryo, in the region where the somites are absent (Fig. 4J-O, *n*=7/7). These data demonstrate that cells forming the anterior somites in wild-type amphioxus embryos integrate the notochord when the FGF signalling pathway is inhibited at the blastula stage, in accordance with the results of the double *in situ* hybridization experiments.

### FGF signalling pathway inhibition induces the loss of the first pair of somites

**Fig. 5.**
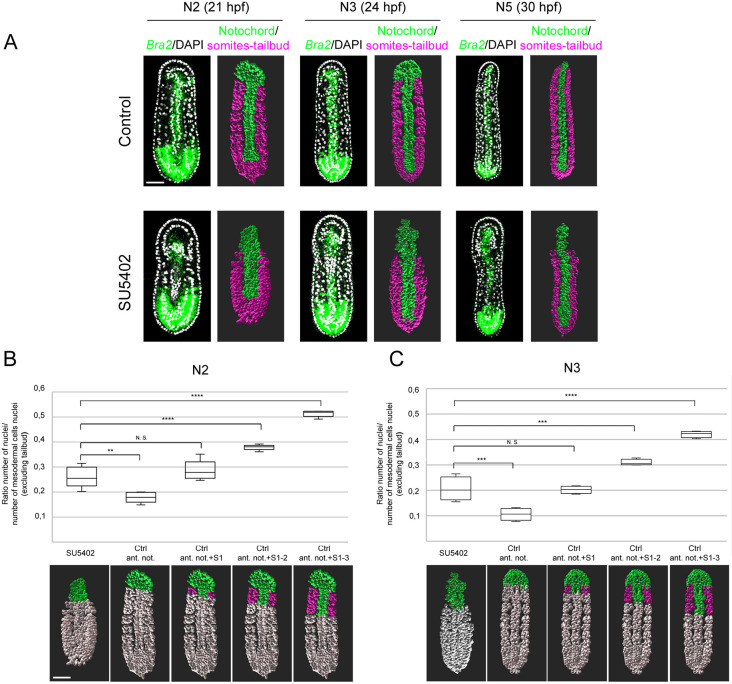
**Only the first somite pair integrates the anterior axial mesoderm after FGFR inhibition.** (A) Confocal images after fluorescent *in situ* hybridization for *Brachyury2* (green) and DAPI staining (white), and corresponding mesodermal nuclei segmentation images of control and SU5402-treated embryos at N2 (21 hpf), N3 (24 hpf) and N5 (30 hpf) stages. The notochord nuclei are in green and the somites and tailbud nuclei are in magenta. Dorsal views with anterior towards the top. Scale bar: 25 µm. (B,C) Graphs presenting the ratio of the number of nuclei of the regions coloured in the schemes presented below per the number of nuclei of the mesoderm (excluding the tailbud *Brachyury2*-expressing region) at N2 (B) and N3 (C) stages. One-way ANOVA and post-hoc Tukey's test analysis results are shown. N.S., non-significant, **P<0.01, ***P<0.001, ****P<0.0001, *n*=4 embryos. Data are median±s.e.m. with the box indicating interquartile range. Scale bar: 25 µm.

Next, we decided to analyse the morphogenesis of the anterior notochord in treated and control embryos and to try to define how many somites were lost under SU5402 treatment. We performed a fluorescent *in situ* hybridization for *Brachyury2*, which is expressed in the notochord and the tailbud at neurula stages (Holland et al., 1995; Somorjai et al., 2008; Yuan et al., 2020), together with nuclei labelling in embryos at N2, N3 and N5 stages (21 hpf, 24 hpf and 30 hpf, respectively). We then used the confocal imaging data to perform embryo 3D reconstructions of nuclei (Fig. 5A, Figs S3 and S4). Each nucleus was manually assigned to a tissue based on *Brachyury2* expression and 3D position (Figs S5 and S6, Movie 1). At the N2 and N3 stages, the notochord cells of the control embryos positioned between the somites have started to intercalate (Fig. 5A). On the other hand, the most anterior *Brachyury2*-expressing cells form a continuous tissue with the endoderm, and correspond to the roof of the anterior mesendoderm. At the N5 stage, the most anterior notochord cells have also intercalated and the first pair of somites has elongated towards the anterior region so that all the notochord cells are positioned between the left and right somites. In SU5402-treated embryos, the *Brachyury2*-expressing region anterior to the first formed somite pair was longer than in control embryos at N2 and N3 stages (Fig. 5A). At the N5 stage, the corresponding cells started to intercalate, and, in contrast to control embryos, we observed a long anterior notochord region that is not flanked by somites. To try to decipher in more detail how many presumptive somites were lost upon treatment, we analysed the ratio of the number of nuclei in the most anterior *Brachyury2*-expressing region (without somites, coloured nuclei in the schemes in Fig. 5B,C) per the total number of nuclei in the notochord+somite territories anterior to the tailbud region in both wild-type and treated embryos. Indeed, in this tailbud region it is hard to assign nuclei to a presumptive structure using exclusively their 3D position. We clearly observed that this ratio was significantly higher in treated embryos compared with controls (Fig. 5B,C, Table S1). We then calculated in control embryos the ratio of the number of nuclei of the most anterior notochord plus the nuclei of the notochord and somite cells corresponding to the first, first plus second or the first three somite pair regions (coloured cells in the schemes of Fig. 5B,C) to the total number of nuclei in the notochord+somite territories anterior to the tailbud (Fig. 5B,C, Table S1). We observed that there was no significant difference between the ratio that included the first somite pair region nuclei in control embryos and the ratio corresponding to the anterior notochord nuclei in treated embryos at the N2 and N3 stages. These data suggest that the presumptive paraxial region that integrates the notochord in SU5402-treated embryos corresponds to the presumptive first somite pair territory. However, as SU5402-treated embryos show a different morphology from controls and a developmental delay at early stages (Fig. S1), we cannot completely rule out the possibility that more somite pairs are lost. On the other hand, the loss of only the first pair after FGF signalling pathway inhibition is consistent with the *in situ* hybridization data, and with somite numbers at later T0 (36 hpf) stage (Fig. S7).

## DISCUSSION

### The anterior tip and the central part of the notochord show different behaviours during amphioxus neurulation: a transient prechordal plate in cephalochordates?

We have shown using 3D reconstructions that, during neurulation, three dorsal axial mesodermal regions can be recognized along the antero-posterior axis of the amphioxus embryo. In the anterior tip, which is small compared with the total length of the embryo at early stages, there are no somites forming and the dorsal axial mesendoderm tissue is continuous with the ventral region that later forms the endoderm at the beginning of neurulation. In the central region, somites, notochord and endoderm segregate, whereas in the posterior region, which forms the so-called tailbud, the different embryonic structures develop sequentially as the embryo elongates. As neurulation proceeds, the cells of the central notochord intercalate earlier than the most anterior notochord cells, as it has been recently highlighted using fine morphometric analyses (Andrews et al., 2021). Our *in situ* hybridization results show that the presumptive central notochord region expresses *Chordin* and *Netrin* together with *Goosecoid* and *ADMP* at the end of gastrulation. However, the anterior dorsal axial mesendoderm expresses *ADMP*, *Lhx2/9a*, *Goosecoid* and *Dmbx* together with *Nodal* and does not express *Chordin* or *Netrin* (Figs 3 and 4). Hence, the most anterior dorsal axial mesendoderm forming the anterior tip of the notochord expresses a different combination of genes from the central notochord. During neurulation, the whole axial mesoderm expresses *Brachyury2* while the expression of *Goosecoid* fades first in the central region, then in the anterior region, and finally becomes restricted to the posterior region (Holland et al., 1995; Neidert et al., 2000; Yasuoka et al., 2019). Thus, *Goosecoid* expression is restricted to axial mesoderm regions in which cells do not intercalate, whereas regions where only *Brachyury2* is expressed coincide with intercalating cell populations. In other words, only when *Goosecoid* expression disappear do notochord cells start to intercalate.

In vertebrates, the axial mesoderm is compartmentalized in two regions. The most anterior region corresponds to the prechordal plate, which derives from ingressing cells of the dorsal organizer that move towards the anterior pole of the embryo during gastrulation. The axial mesoderm posterior to the prechordal plate forms the notochord plate and further differentiates into the notochord. Similar to the amphioxus anterior notochord cells at gastrula and early neurula stages, the vertebrate prechordal plate cells do not intercalate, but, contrary to amphioxus, in which these cells intercalate during neurulation when *Goosecoid* expression disappears, in vertebrates these cells never intercalate. Interestingly, the vertebrate prechordal plate expresses *Gsc1* (the ortholog of *Goosecoid* in amphioxus) in all vertebrates studied (Cho et al., 1991; Blum et al., 1992; Izpisúa-Belmonte et al., 1993; Schulte-Merker et al., 1994), whereas the notochord plate and notochord express *Brachyury* orthologs (Wilkinson et al., 1990; Smith et al., 1991; Schulte-Merker et al., 1994; Kispert et al., 1995), with the expression patterns of both genes becoming exclusive after gastrulation (De Robertis et al., 1994). *Gsc1* has been described as a repressor of *Brachyury* expression (Artinger et al., 1997) and it has recently been shown that *Gsc1* inhibits the Wnt/PCP-mediated convergent extension of the notochord in mouse and *Xenopus* (Ulmer et al., 2017).

A comparison between these vertebrate data and what we observed in amphioxus, suggests that, during amphioxus gastrulation and early neurulation, *Goosecoid* may inhibit the intercalation of dorsal axial mesendoderm cells. While neurulation proceeds, *Goosecoid* expression decreases in the central region of the axial mesoderm that expresses *Brachyury2*, allowing convergent extension. Then, at later neurulation stages, the loss of *Goosecoid* expression in the anterior axial mesoderm expressing *Brachyury2* leads to a delayed convergent extension behaviour compared with central axial cells. This would contribute to the late elongation of the notochord in the anterior region of amphioxus, resulting in a notochord that grows anterior to the central nervous system, which is a specific feature of cephalochordates.

It has been suggested that amphioxus does not have a prechordal plate (Neidert et al., 2000). However, recent data highlighted the peculiarity of the anterior axial mesoderm in terms of cell behaviour (Andrews et al., 2021) and of gene expression (Albuixech-Crespo et al., 2017), leading to the proposition of the existence of a ‘prechordal process’ in amphioxus (Ferran et al., 2022). In line with this suggestion, we propose that amphioxus possesses a structure similar to the vertebrate prechordal plate but only transiently during early embryonic development and that the differences observed in the behaviour of the anterior axial mesodermal tissue between amphioxus and vertebrates are due to the loss of *Goosecoid* expression during neurulation in the anterior axial mesoderm that expresses *Brachyury2*. The anteriorly growing notochord has been proposed to be a derived feature of amphioxus (Chen et al., 1995; Holland et al., 1995). Here, we suggest that this derived characteristic could result from the duplication of the *Brachyury* gene in the cephalochordate lineage. Indeed, there are two *Brachyury* genes in all cephalochordates studied so far that arose by tandem duplication (Inoue et al., 2017). *Brachyury2* is expressed in the whole axial mesoderm and in the paraxial region of the tailbud during neurulation (Holland et al., 1995; Yuan et al., 2020). On the other hand, *Brachyury1* is expressed in the notochord region, with the exception of the anterior part, where *Brachyury2* is expressed at the time at which *Goosecoid* expression fades (Yuan et al., 2020). We could hence conceive that the ancestral chordate expression pattern of *Brachyury* was similar to what is observed in vertebrates, and that *Brachyury1* lost its expression in the somitic tailbud region, whereas *Brachyury2* acquired a new anterior domain of expression in the *Goosecoid*-expressing transient prechordal plate, which is responsible for its convergent extension behaviour.

### FGF signalling pathway inhibition induces a change of fate of the anterior paraxial mesoderm territory corresponding to the presumptive first somite pair

Our cell-tracing experiments showed that the presumptive anterior paraxial mesoderm cells integrate the notochord when the FGF signalling pathway is inhibited during gastrulation in amphioxus. The *in situ* hybridization data indicate that these cells express *Goosecoid* in SU5402-treated embryos, and that the *Goosecoid*-expressing region corresponds to the region expressing *Dmbx*, which forms the first somite pair in the wild-type amphioxus embryo. Together with the morphological analysis, these expression data suggest that the cells of the region corresponding to the first somite pair change their fate towards the transient prechordal plate fate. This result refutes our previous proposition based on the initial analysis of SU5402-treated embryos in which we claimed that the first three somite pairs were lost under FGF signalling pathway inhibition (Bertrand et al., 2011). This assertion was based on several observations and on the fact that in the late neurula T0 stage treated embryos, the anterior notochord, in the region where no somites are observed, is much longer than in controls and its length approximately corresponds to the length of the notochord normally flanked by the first three somite pairs (Bertrand et al., 2011, 2015). However, the data presented in this work explain this discrepancy, which would be due to the fact that the presumptive paraxial mesoderm cells seem to acquire a prechordal fate and not a notochordal fate. Hence, at early developmental stages the region that is affected by the FGFR inhibitor treatment is not long, but, as neurulation proceeds, the intercalation of these cells make the affected territory seemingly much longer in proportion to the whole antero-posterior axis of the body.

The first somite pair of amphioxus shows characteristics that differentiate them from all the other somites. At the morphological level, they elongate anteriorly and have a different shape compared with posterior somites (Conklin, 1932). Moreover, their ventral domain give rise to specific structures. The non-myotome part of the first left somite forms the Hatschek's nephridium, the excretory organ of the larva and the oral mesoderm, which is associated to the mouth opening process on the left side (Holland, 2018). In addition, both left and right first somites express genes orthologous to vertebrate hematopoietic genes during neurulation (Pascual-Anaya et al., 2013). At earlier developmental stages, the unique characteristics of the first somite pair are the absence of expression of any Wnt ligand, whereas all the other somites express specific combinations of Wnt genes (Somorjai et al., 2018), and the expression of *Cerberus*, which becomes restricted to the right first somite at the end of gastrulation and disappears in the mesoderm during neurulation (Le Petillon et al., 2013). Our data provide further support for these differences between the first somite pair and the others by demonstrating a distinct requirement of the FGF signal for their formation.

In vertebrates, the different initial fates of mesodermal cells are acquired through the interaction of various signalling pathways acting during gastrulation, which, unlike in amphioxus (Zhang et al., 1997), is a developmental process associated with important cell movements. During this period, BMP acts as a ventralizing signal, Nodal as a dorsalizing signal, and FGF and Wnt cooperate to specify an intermediate dorso-lateral fate in the forming mesoderm (Tuazon and Mullins, 2015). Enhanced FGF signal was shown, for example, to expand the paraxial mesoderm ventrally in the zebrafish embryo (Furthauer et al., 1997). On the other hand, the expression of the dominant-negative FGF receptor XFD in *Xenopus* leads to the absence of muscle and notochord cells, although the most anterior axial mesoderm, which expresses *Gsc1*, is not affected, highlighting different FGF requirements of the prechordal plate and the notochord plate (Amaya et al., 1993). The use of the SU5402 inhibitor in *Xenopus* allowed the refinement of this view, showing that FGF signal is required for paraxial mesoderm initial specification and for axial mesoderm maintenance (Fletcher and Harland, 2008). In amphioxus, we showed that FGF signal is dispensable during gastrulation for mesoderm formation; however, it is required to specify the paraxial domain corresponding to the first somite pair region. Which signal operates to define the paraxial domain posteriorly is still unknown, although Wnt is a good candidate.

### Evolution of the vertebrate head: a refined scenario

**Fig. 6.**
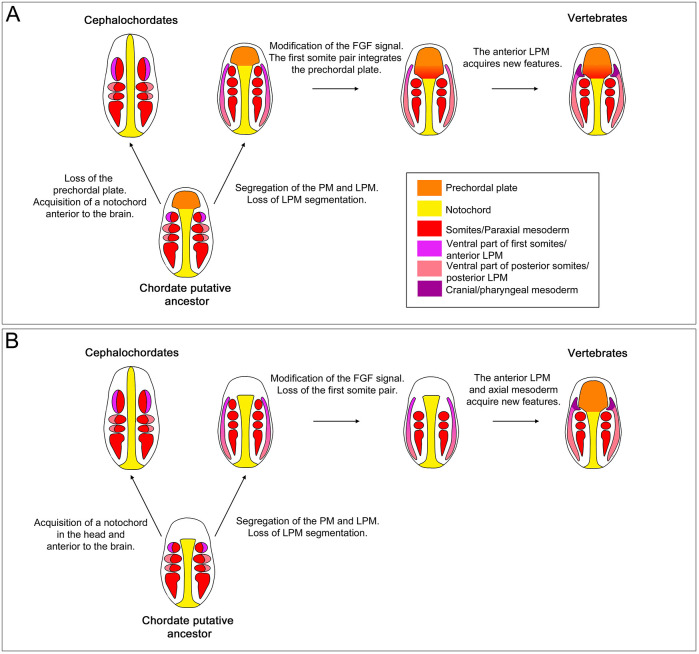
**Two scenarios for the evolution of the anterior mesoderm in chordates.** (A,B) Schematics of embryos at the early somitogenesis stage are presented as dorsal views with anterior towards the top. Arrows correspond to evolutionary steps. Colour coding of different mesodermal territories is shown in the key. PM, paraxial mesoderm; LPM, lateral plate mesoderm. (A) In this scenario, we considered that the chordate ancestor had a prechordal plate-like structure, as observed transiently in amphioxus. (B) In this scenario, we considered that the chordate ancestor had neither an anterior notochord nor a prechordal plate.

Based on our previous work we formulated a multistep hypothetical evolutionary scenario underlying vertebrate head mesoderm origin in which the chordate ancestor had a paraxial mesoderm segmented all along its antero-posterior axis, as observed in extant cephalochordates (Bertrand et al., 2011; Aldea et al., 2019). Segregation of the lateral/ventral mesoderm from the paraxial mesoderm, associated with a loss of segmentation of the lateral/ventral mesoderm, was the first step (Aldea et al., 2019). The second step was the regionalization of the lateral/ventral mesoderm. Finally, in a third step, the loss of the anterior paraxial mesoderm, probably through a modification of the role of the FGF signalling pathway, would have reduced the developmental constraints imposed by the somites and allowed the lateral plate mesoderm to ‘colonize’ this anterior dorsal territory for the formation of a ‘novel’ muscular system. The present study allows us to refine this hypothesis, by including a scenario for the evolution of the prechordal plate. Regarding the axial mesoderm, although it is accepted that the ancestral chordate possessed a notochord, which is a synapomorphy for this group (Annona et al., 2015), it is still unclear whether it ran along its entire antero-posterior axis. Indeed, each group of extant chordates shows a different pattern. In cephalochordates, the notochord extends from the anterior of the cerebral vesicle to the posterior end of the animal. In tunicates, the notochord is present only in the tail, whereas in vertebrates the anterior axial mesoderm forms the prechordal plate anterior to the otic vesicle. The fossil record can provide some support, although only larval or adult stages are available, and the interpretation in terms of structures observed and phylogenetic position of the species described is subject to much debate (Holland and Chen, 2001; Swalla and Smith, 2008). Hence, in the two fossil species that can be recognized as potential early Cambrian chordates, *Pikaia gracilens* and *Yunnanozoon lividum* (Chen et al., 1995; Morris and Caron, 2012), there is no notochord in the anterior region. This suggests that the ancestral chordate had a prechordal plate-like structure in the anterior region, or that it had neither anterior notochord nor prechordal plate. If we consider that the ancestral chordate had a prechordal plate-like anterior axial mesoderm, which is the most parsimonious hypothesis if we consider that cephalochordates possess a transient prechordal plate as we and others suggest (Albuixech-Crespo et al., 2017; Andrews et al., 2021; Ferran et al., 2022), we can propose a new evolutionary framework for the appearance of the complex vertebrate head (Fig. 6A). Thus, during vertebrate evolution, the role of the FGF signal changed, resulting in the acquisition of new functions and the loss of its contribution to the anterior mesoderm formation, with similar effects to those we observed in amphioxus after FGFR inhibition. This would have led to the formation of a bigger prechordal plate that is not flanked by somites but by the anterior lateral plate mesoderm. The latter would have lost the constraints imposed by the neighbouring somites and gained the ability to form new tissues, among which are the muscles derived from the cranial/pharyngeal mesoderm. In this scenario, the enlargement of the prechordal plate territory and the inclusion of the most anterior paraxial mesoderm in this structure could explain the larger anterior brain observed in vertebrates versus non-vertebrate chordates. It could also explain the capacity of the prechordal plate to form some head muscles. Indeed, the anterior brain of vertebrates is induced by the prechordal plate, which is sometimes described as the ‘head organizer’ in non-amniote vertebrates (De Robertis et al., 1994), and the prechordal plate gives rise to the extraocular muscles in vertebrates (Noden and Francis-West, 2006). In this scenario, cephalochordates would have also derived from the ancestral state, with the acquisition of a notochord growing anteriorly. This cephalochordate synapomorphy would have resulted from the duplication of the *Brachyury* gene and gain of expression of one of the duplicates in the anterior axial mesoderm. However, if we consider that the chordate ancestor did not have a prechordal plate-like structure or a notochord, we can draw another hypothetical scenario (Fig. 6B). Thus, cephalochordates would have acquired an anterior notochord, whereas, during vertebrate evolution, the loss of the anterior segmented paraxial mesoderm and associated developmental constraints would have led to the remodelling of the axial and lateral anterior mesoderm territories. This would result in the appearance of the prechordal plate and of the cranial/pharyngeal mesoderm.

To conclude, by studying the fate of the anterior paraxial mesoderm after FGF signalling pathway inhibition in cephalochordates, we highlighted the peculiarities of the anterior notochord in this chordate lineage and provide new insights into how the complex head of vertebrates, with a large brain and specific muscles, might have emerged during evolution.

## MATERIALS AND METHODS

### Embryo manipulation

Ripe adults of *Branchiostoma lanceolatum* were collected at the Racou beach near Argelès-sur-Mer, France (latitude 42° 32′ 53′′ N, longitude 3° 03′ 27′′ E). Gametes were obtained by heat stimulation as previously described (Fuentes et al., 2007) and staging is as described previously (Bertrand et al., 2021; Carvalho et al., 2021). Before pharmacological treatment, embryos were transferred to scraped Petri dishes filled with filtered seawater to prevent the eggs from sticking to the plastic. InSolution SU5402 (572631; Sigma-Aldrich) at 10-2 M was added to cultures of embryos to a final concentration of 25 µM at the blastula stage [B stage, 5 h post-fertilization (hpf) at 19°C] and embryos were raised in this medium until fixation. Control embryos were raised simultaneously with equivalent concentrations of DMSO in filtered seawater. Embryos were fixed in paraformaldehyde (PFA) 4% in MOPS buffer, dehydrated in 70% ethanol and then kept at −20°C. Staging is according to Carvalho et al. (2021).

### TUNEL assay

Apoptotic cells were detected using the Click-iT Plus TUNEL Assay for *In Situ* Apoptosis Detection, Alexa Fluor 647 dye kit (C10619; ThermoFisher Scientific) following supplier instructions, with some modifications. Embryos stored in 70% ethanol were rehydrated in 1×PBS (phosphate-buffered saline). Embryos were incubated in 0.5% Triton X-100 in PBS for 1 h, digested with 1× Proteinase K solution (from the manufacturer kit) for 15 min at 37°C, and then washed in PBT (1×PBS and 0.1% Tween20). For positive controls, a DNase I treatment (10 µg/ml) was undertaken for 30 min at room temperature prior to the labelling procedure. All the embryos were post-fixed in 4% PFA in PBS for 20 min, washed in PBS and rinsed with deionized water. A terminal deoxynucleotidyl transferase (Tdt) reaction was carried out according to the manufacturer's instructions for 120 min at 37°C. The reaction was terminated by rinsing the embryos in deionized water, followed by washes in 1×PBS. A Click-iT Plus reaction was performed following the manufacturer's instructions, and the embryos then washed with 3% BSA (bovine serum albumin) in PBS for 5 min. For imaging, embryos were embedded into ProLong Diamond Antifade Mountant with DAPI (P36962; ThermoFisher Scientific) for 24 h. Confocal stacks (1 µm slice/150 µm deep) were generated on whole embryos on a Leica SP8 confocal microscope using a 40× oil-immersion objective.

### Photoconvertible Kaede experiment

Kaede coding DNA sequence from *Trachyphyllia geoffroyi* was cloned into the pCS2+ expression vector backbone (a gift from Agnes Roure, Sorbonne Université, Banyuls-sur-Mer, France). The vector was linearized and *in vitro* transcription was performed using the mMESSAGE mMACHINE SP6 Transcription Kit (ThermoFisher Scientific). Microinjection of the mRNA was carried out as described previously (Hirsinger et al., 2015). At 10 hpf (G4), embryos were individualized in confocal glass-bottom culture dishes. Embryos were positioned in a blastopore view in order to proceed to the photoconversion. Kaede photoconversion and imaging was undertaken on a Leica SP8 confocal microscope using a 20× oil-immersion objective. Photoconversion was carried out using the FRAP module from the Leica software. At 30 hpf, full *z*-stacks (1 μm slice/50 μm deep) were acquired using a 40× oil-immersion objective for each photoconverted embryo after immobilization into 2× sea water for 30 s (1038.4 mM of NaCl, 22.2 mM of KCl, 20 mM of CaCl_2_-2H_2_0, 49 mM of MgCl_2_-6H_2_0 and 51 mM of MgSO_4_-7H_2_0).

### Fluorescent *in situ* hybridization

Whole-mount *in situ* hybridization probes were synthesized using the DIG and Fluorescein labelling system (Roche) after plasmid linearization with the appropriate enzymes. Double fluorescent *in situ* hybridizations were performed as described previously (Coulcher et al., 2020), with some modifications. Embryos were washed with the TNT solution [100 mM Tris (pH 7.5); 150 mM NaCl; 0.1% Tween20) for 1 h (4×15 min) after post-antibody washes for the revelation of the DIG probe. A 30 min incubation with TSA was performed at a final concentration of 1:100 in amplification diluent buffer (NEL753001KT, Perkin Elmer) followed by three washes in TNT. The peroxidase was inactivated by a 10 min incubation in 2% H_2_0_2_ in PBT. Embryos were then incubated in anti-fluorescein antibody overnight at 4°C. The same steps were applied for the revelation of the fluorescein probe for double labelling. For imaging, embryos were embedded into ProLong Diamond Antifade Mountant with DAPI (P36962; ThermoFisher Scientific) for 24 h. Images were acquired on a Leica SP8 Confocal microscope using a 40× oil-immersion objective. The images were analysed using Fiji for the double *in situ* hybridization (Schindelin et al., 2012). The reconstruction of the different populations of nuclei was undertaken manually using Imaris 8.3 analysis software (Bitplane, Switzerland). The accession numbers of the sequences used for probe synthesis are given in Table S2.

### Statistical analysis

Statistical tests were performed using R. First, a Shapiro-Wilk test and Levene test were performed to test for the normal distribution of the data and the homogeneity of variance for each considered group. We then undertook a one-way ANOVA to test whether the means of the measurement variables in the groups were significantly different, followed by a post-hoc Tukey's test.

## Supplementary Material

Supplementary information

Reviewer comments
