# Supplementary material for: Functions of the FGF signalling pathway in cephalochordates provide insight into the evolution of the prechordal plate
Source: Development. 2022 May 16;149(10):dev200252. doi: 10.1242/dev.200252 (PMC9188755; doi:10.1242/dev.200252)
Supplement: Supplementary information [file develop-149-200252-s1.pdf]

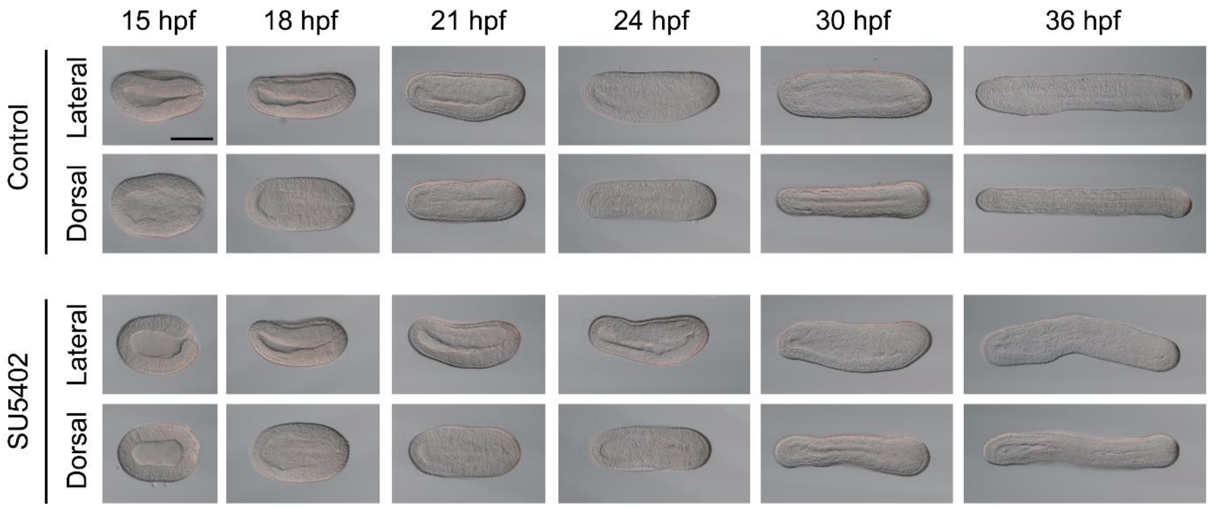

Fig. S1. DIC microscopy images of control and SU5402-treated embryos fixed at 15 hpf (G6), 18 hpf (N1), 21 hpf (N2), 24 hpf (N3), 30 hpf (N5) and 36 hpf (T0). Lateral and dorsal views are shown with anterior to the left and dorsal to the top for lateral views. Scale bar : 50 µm.

Development • Supplementary information

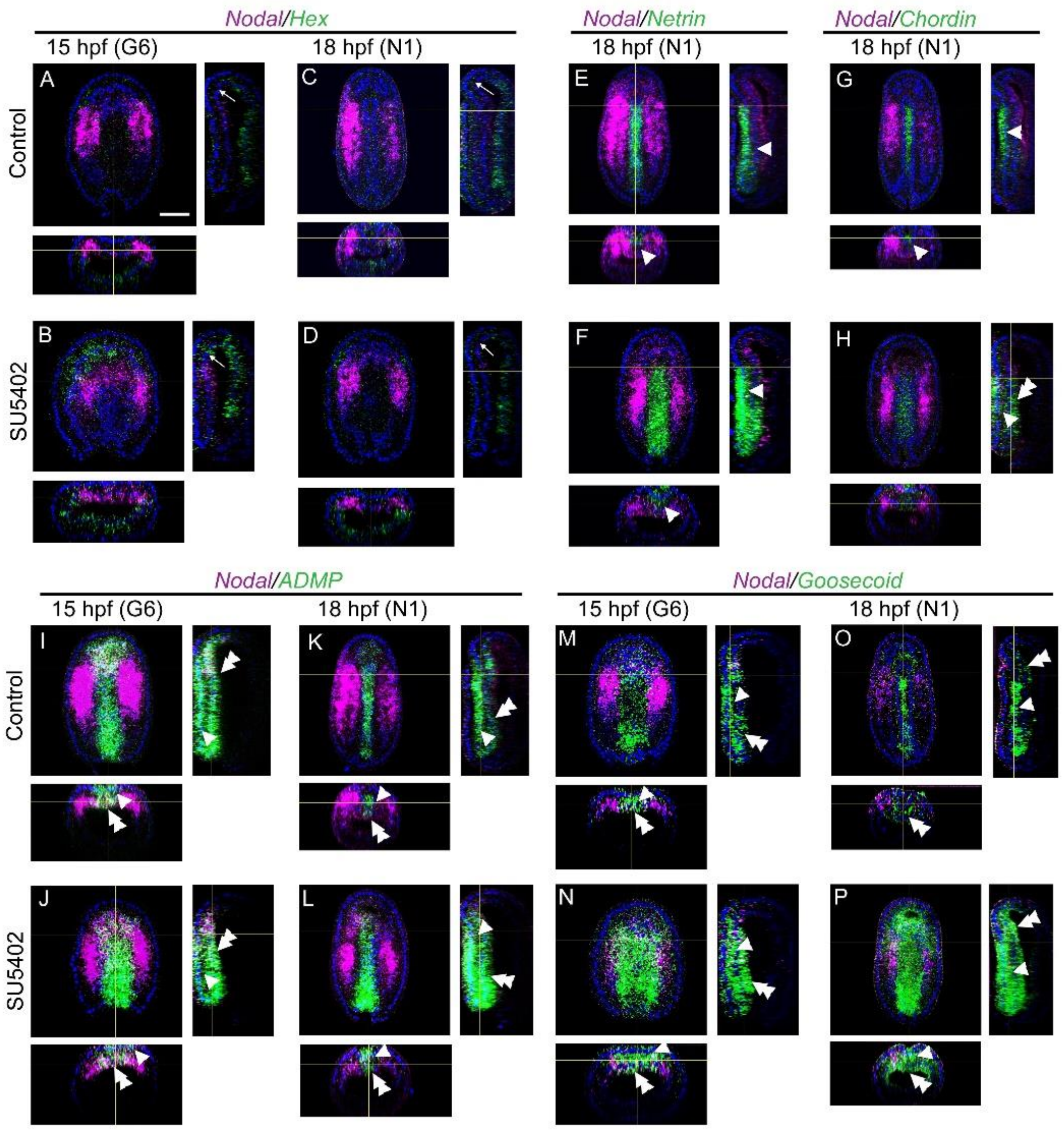

Fig. S2. Additional orthogonal views of double *in situ* hybridization for *Nodal* and *Netrin, Chordin, ADMP* and *Goosecoid.* Double *in situ* hybridization of *Nodal* (magenta) together with *Hex*, *Netrin, Chordin, ADMP* and *Goosecoid* (green) at 15 hpf (G6) and/or 18 hpf (N1) stages in control embryos and embryos treated with SU5402. Orthogonal views (XZ below the main panel, YZ on the right) of stacks are shown. Main panels are dorsal views with anterior to the top. White arrows point the frontier between *Hex* and *Nodal* expression territories, arrowheads point the neural plate layer, and white double arrowheads the dorsal axial mesendoderm. Scale bar : 50µm.

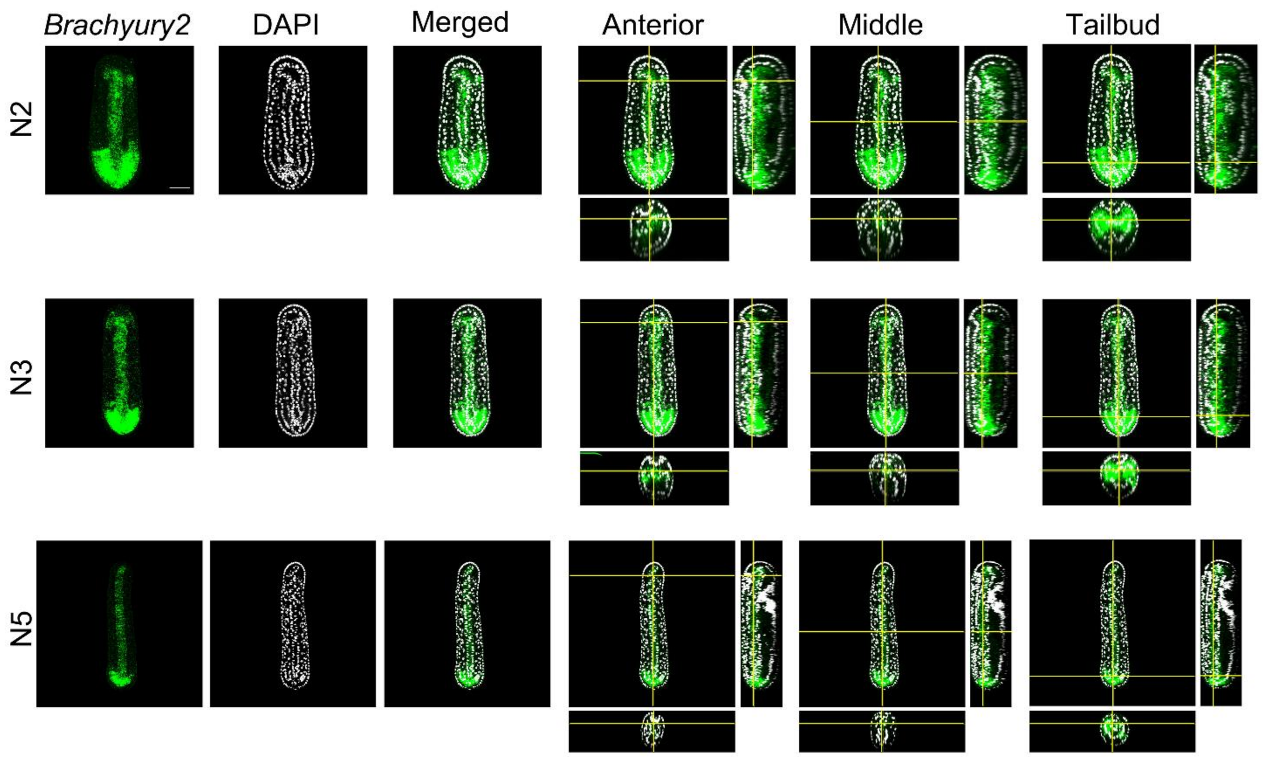

Fig. S3. *Brachyury2 in situ* hybridization (green) and nuclei labelling (DAPI, white) in control embryos at 21 hpf (N2), 24 hpf (N3) and 30 hpf (N5) stages. Orthogonal views of stacks are shown with transverse sections at the anterior, middle and tailbud levels. Anterior to the top and dorsal to the left for sagital sections and to the top for transverse sections. Scale bar : 50µm**.**

Development • Supplementary information

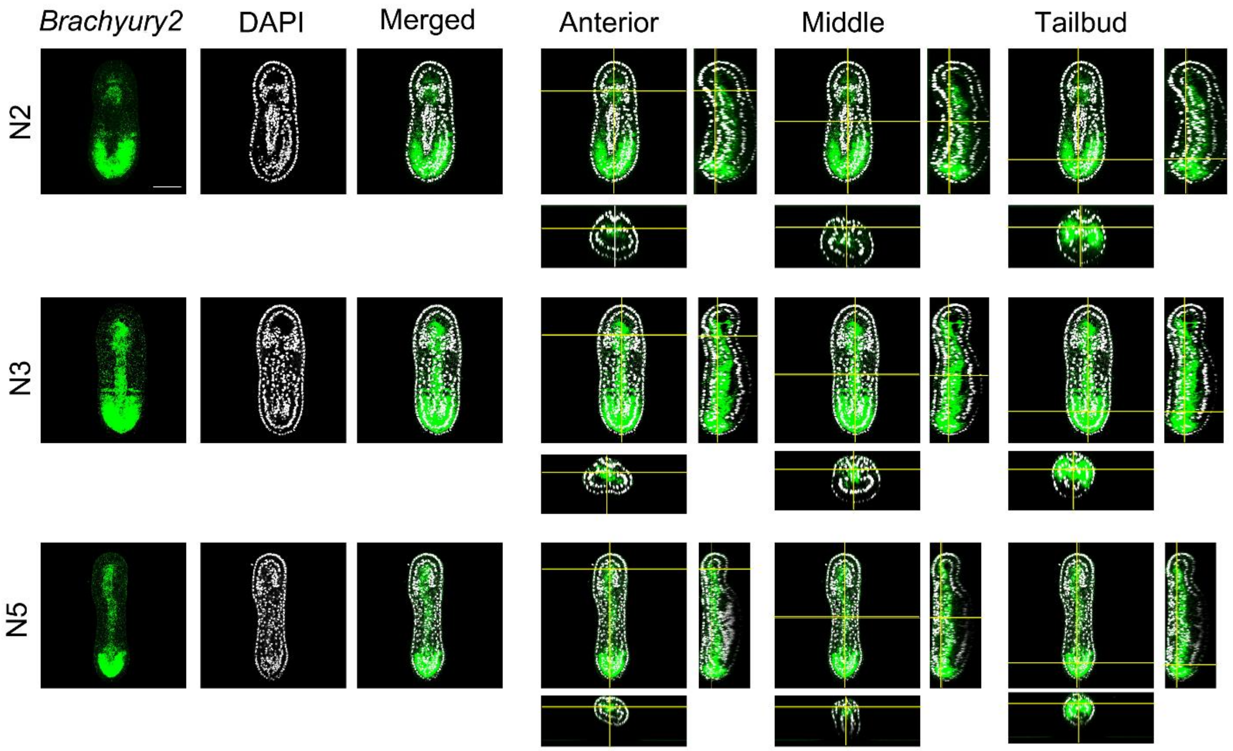

Fig. S4. *BRA2 in situ* hybridization (green) and nuclei labelling (DAPI, white) in SU5402-treated embryos at N2, N3 and N5 stages. Orthogonal views of stacks are shown with transverse sections at the anterior, middle and tailbud levels. The yellow lines indicate the position of the different optical sections. Anterior to the top and dorsal to the left for sagital sections and to the top for transverse sections. Scale bar: 50µm**.**

Development • Supplementary information

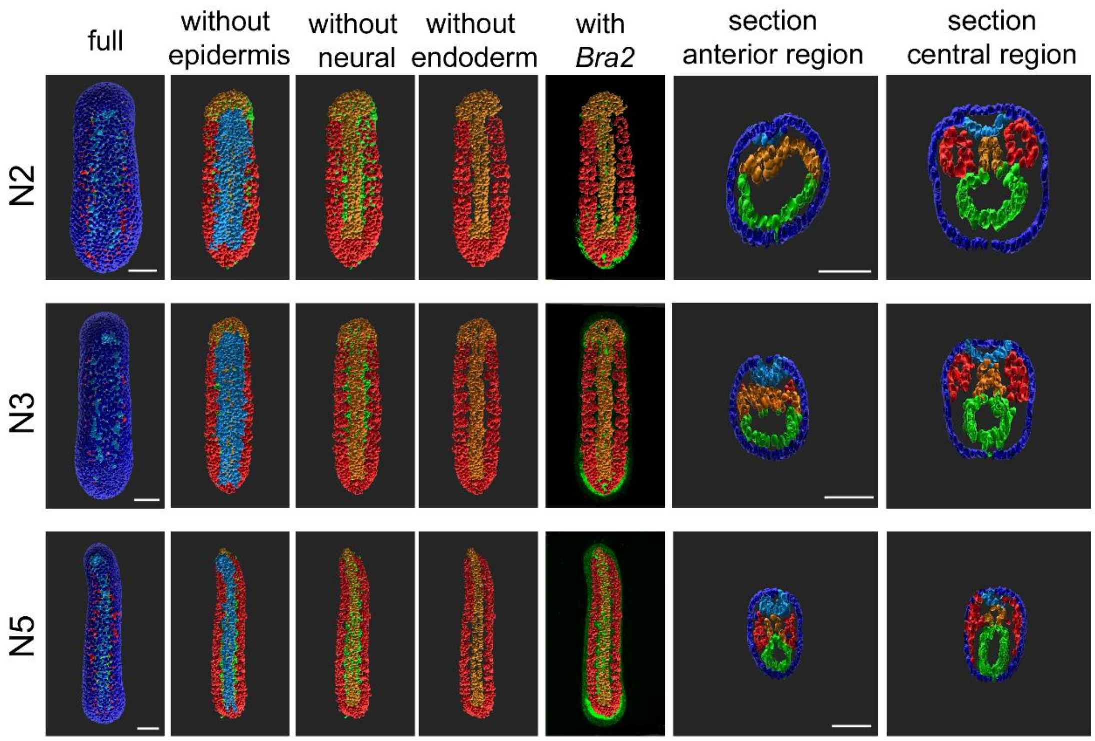

Fig. S5. Embryo nuclei 3D reconstruction from confocal images after *Brachyury2 in situ* hybridization and DAPI labelling. One control embryo is presented for 21 hpf (N2), 24 hpf (N3) and 30 hpf (N5) stages. The nuclei were colored according to the *Brachyury2 (BRA2) in situ* hybridization labelling and to their 3D position. Blue: epidermis; light blue: neural plate/tube; green: endoderm; orange: notochord; red: somites and tailbud. Transverse sections at the anterior and central level are presented on the right. Anterior to the top for dorsal views and dorsal to the top for sections. Scale bar: 25µm.

Development • Supplementary information

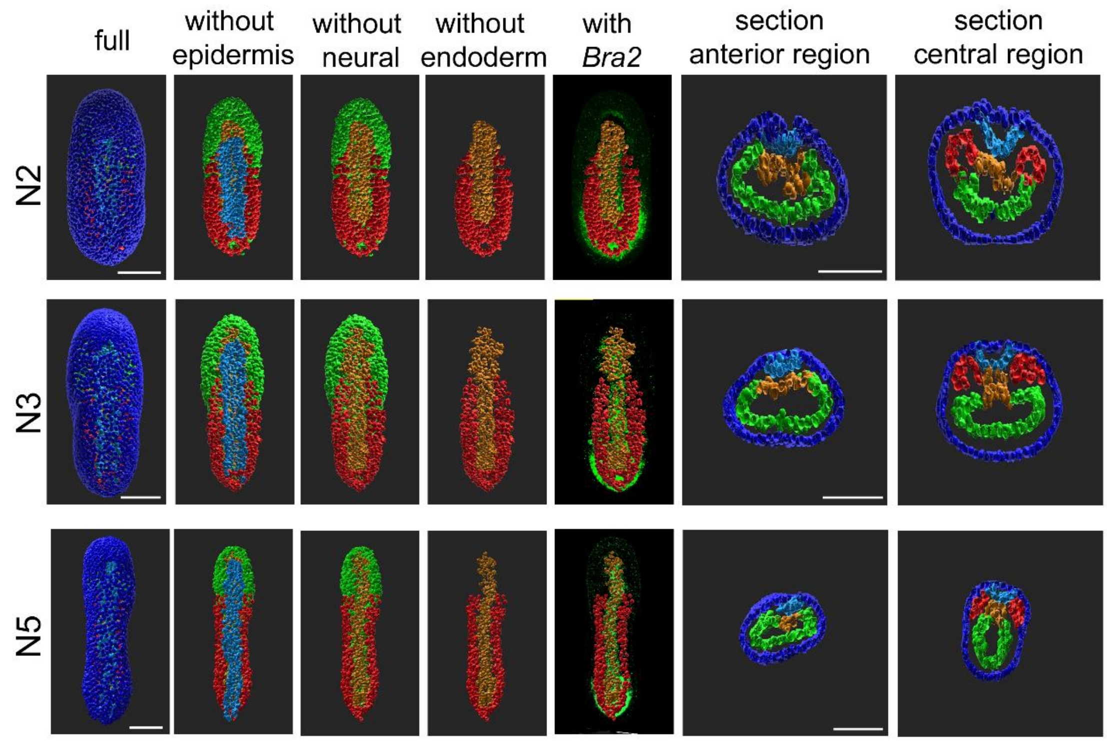

Fig. S6. Embryo nuclei 3D reconstruction from confocal images after *Brachyury2 in situ* hybridization and DAPI labelling. One SU5402-treated embryo is presented for 21 hpf (N2), 24 hpf (N3) and 30 hpf (N5) stages. The nuclei were colored according to the *Brachyury2 (Bra2) in situ* hybridization labelling and to their 3D position. Blue: epidermis; light blue: neural plate/tube; green: endoderm; orange: notochord; red: somites and tailbud. Transverse sections at the anterior and central level are presented on the right. Anterior to the top for dorsal views and dorsal to the top for sections. Scale bar: 25µm.

Development • Supplementary information

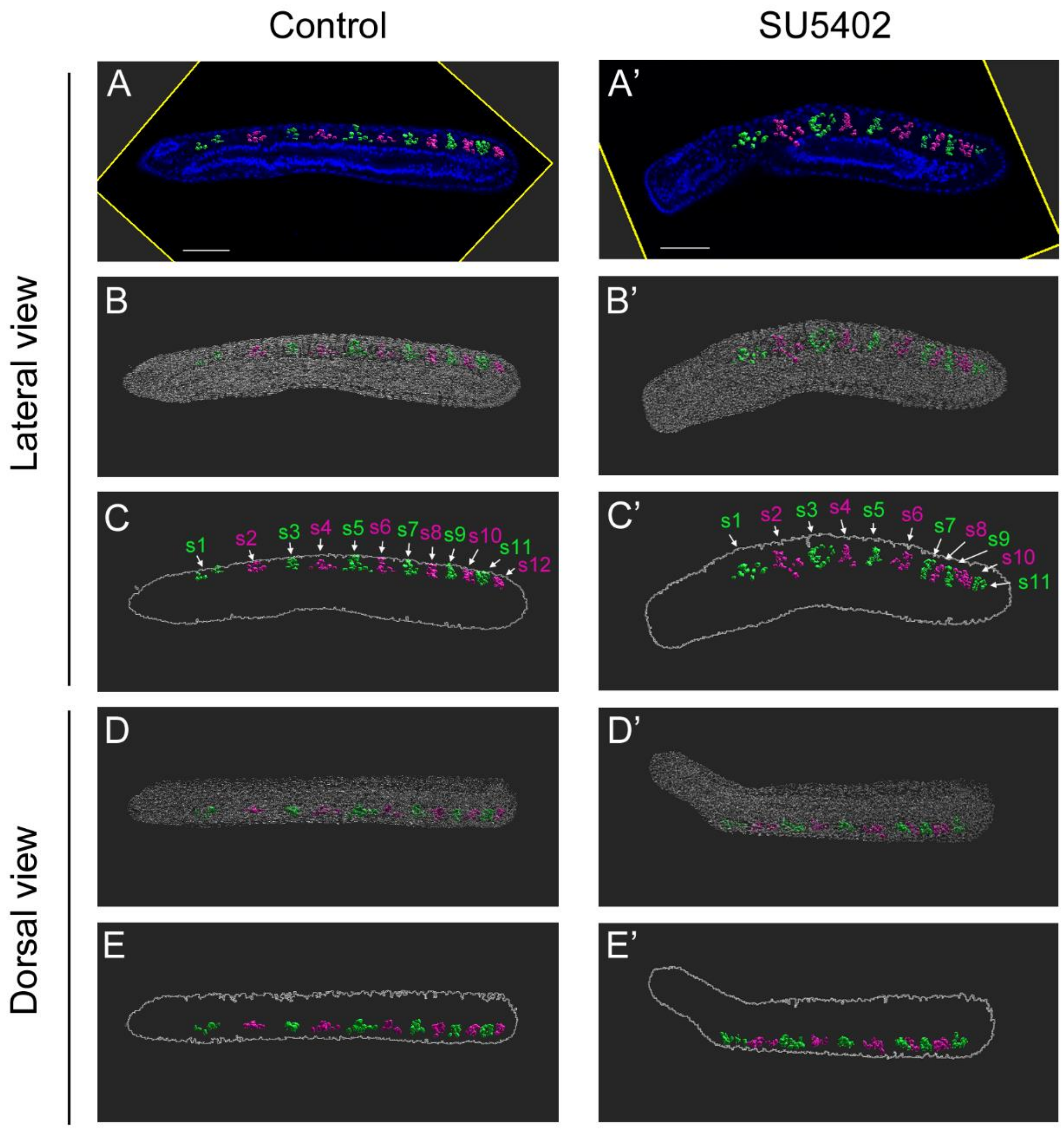

Fig. S7. Somites counting at the T0 stage. Nuclei of a control embryo (A-E) and a SU5402-treated embryo (A'-E') were segmented after DAPI labelling and confocal imaging. The nuclei were assigned to somites given their 3D position. (A-A') Snapshots showing one optic section superposed with the somite nuclei. (B-B') Pictures showing all the nuclei (transparent) and the somite nuclei (in color) in a lateral view corresponding to the view in (A-A'). (C-C') Somite nuclei are shown as in (B-B') as well as the embryo outline. Twelve somites are observed in the control embryo and eleven in the SU5402-treated embryo. (D-D') Pictures showing all the nuclei (transparent) and the somite nuclei (in color) in a dorsal view. (E-E') Somite nuclei are shown as in (D-D') as well as the embryo outline. Scale bar : 50 µm.

Table S1. Nuclei counting and statistics.

**Counting**												
Timing	Treatment	Mesodermal cells except tailbud	Bra2-positive cells anterior to the first somite pair in SU5402 embryos	Bra2-positive cells anterior to the first somite pair in control embryos	Notochord cells+first somite pair	Notochord cells+two first somite pairs	Notochord cells+three first somite pairs	Ratio anterior notochord in SU5402 embryos	Ratio anterior notochord in control embryos	Ratio notochord cells+first somite pair in control embryos	Ratio notochord cells+two first somite pairs in control embryos	Ratio notochord cells+three first somite pairs in control embryos
21hpf	Control	692		136	243	262	355		0,19653179	0,35115607	0,37861272	0,51300578
21hpf	Control	559		83	138	219	292		0,14847943	0,24686941	0,39177102	0,52236136
21hpf	Control	665		119	185	253	345		0,17894737	0,27819549	0,38045113	0,51879699
21hpf	Control	604		121	175	230	316		0,20033113	0,2897351	0,3807947	0,52317881
21hpf	Control	765		130	201	276	376		0,16993464	0,2627451	0,36078431	0,49150327
21hpf	SU5402	259	64					0,24710425				
21hpf	SU5402	316	90					0,28481013				
21hpf	SU5402	272	55					0,20220588				
21hpf	SU5402	264	83					0,31439394				
21hpf	SU5402	314	80					0,25477707				
24hpf	Control	743		98	156	225	317		0,13189771	0,20995962	0,30282638	0,42664872
24hpf	Control	725		56	134	217	292		0,07724138	0,18482759	0,29931034	0,40275862
24hpf	Control	823		79	162	253	356		0,09599028	0,19684083	0,30741191	0,43256379
24hpf	Control	822		96	179	269	345		0,11678832	0,21776156	0,32725061	0,41970803
24hpf	SU5402	370	98					0,26486486				
24hpf	SU5402	425	79					0,18588235				
24hpf	SU5402	445	96					0,21573034				
24hpf	SU5402	516	80					0,15503876				

**Tukey test 21 hpf**							**Tukey Test 24 hpf**			
Group 1	Group 2	estimate	conf. low	conf. high	p.adj		estimate	conf. low	conf. high	p.adj
Ratio anterior notochord in control embryos	Ratio anterior notochord in SU5402 embryos	0,0818134	0,0275257	0,1361011	0,00178		0,0998997	0,0438836	0,1559157	0,000495
Ratio anterior notochord in control embryos	Ratio notochord cells+first somite pair in control embryos	0,1068954	0,0526076	0,1611831	0,0000815		0,096868	0,0408519	0,152884	0,000674
Ratio anterior notochord in control embryos	Ratio notochord cells+two first somite pairs in control embryos	0,1996379	0,1453502	0,2539256	0		0,2037204	0,1477043	0,2597365	0,0000001
Ratio anterior notochord in control embryos	Ratio notochord cells+three first somite pairs in control embryos	0,3349244	0,2806367	0,3892121	0		0,3149404	0,2589243	0,3709564	0
Ratio anterior notochord in SU5402 embryos	Ratio notochord cells+first somite pair in control embryos	0,25082	-0,0292057	0,0793697	0,0645		-0,0030317	-0,0590478	0,0529844	1
Ratio anterior notochord in SU5402 embryos	Ratio notochord cells+two first somite pairs in control embryos	0,1178245	0,0635368	0,1721122	0,0000224		0,1038207	0,0478047	0,1598368	0,000334
Ratio anterior notochord in SU5402 embryos	Ratio notochord cells+three first somite pairs in control embryos	0,253111	0,1988233	0,3073987	0		0,2150407	0,1590246	0,2710568	0
Ratio notochord cells+first somite pair in control embryos	Ratio notochord cells+two first somite pairs in control embryos	0,0927425	0,0384548	0,1470303	0,000458		0,1068524	0,0508363	0,1628685	0,000247
Ratio notochord cells+first somite pair in control embryos	Ratio notochord cells+three first somite pairs in control embryos	0,228029	0,1737413	0,2823167	0		0,2180724	0,1620563	0,2740885	0
Ratio notochord cells+two first somite pairs in control embryos	Ratio notochord cells+three first somite pairs in control embryos	0,1352865	0,0809987	0,1895742	0,0000031		0,11122	0,0552039	0,1672361	0,000161

Development • Supplementary information

Table S2. Accession numbers of sequences used for *in situ* hybridization probe synthesis.

Gene Name	Accession number/sequence
ADMP	ACTAGTGATTCTAGTGACAGCCGCAGTTTGCTGCTACCATATCGTCATACTGTTTC<br>AATATAACGTTTTCTTCGTCGTCGAAGTAGAGCAGGTTGATGGAGTAGAGTTTGTT<br>GGGTACGCAGCATGGCTGGCCCACGTCTTCTTGTAACCTTAGCGCGTTCATGATGG<br>ACTGAACCGTGGCGTGGTTGGTGGGTTTCTGAGACTGGCCCAAGGGGAACGGACAC<br>TTGCCCTTGCAGTGGTAGGCGTTGTAGCCTTTAGGGGAGATGATCCATCCCGACCA<br>GCCAATGGCGTCAAAGTCCACGTACAAGTTGCGTCTAGTGCACGGCTCGAATTGTC<br>TGTTAGTCGGGTACCGCTGAGCCGCCCTCTCGACCCGCGTTCTGTTGTTTTTCCTG<br>TTCGCTGATTTCTCCACCGTGTTTGTCCTTTCGTTTCGCCACTGCTGTTGTGTCTC<br>AGGGCTTGGGTCATAGTAGTTGTTCAAGATTTCTTTGTTTGTCGACGTGCTGTCGT<br>GGTAATCGTAGTCTTCACCGTTACTGGGAGCACTTTCATAGGTGGCTGCGGATGCT<br>GATCTCGGTCTGCCGTCGTCACTAAACAGAACGAGGATTGGTTCCTTACTTCTGTG<br>GTGCTCCTTCCTTTTGGCGAATCTGATGACAGTCTGGTCGAGGCTGCTCCCCGTCA<br>AGGACGCTATCGTGACCAGCAGGCCGAAGTTGGCGTTCTTGTCAGCGACCCAGTCC<br>TGCACCGCAGGCTTGATGTTGAAGACCTCCCAGCCGGAGCCGTGCAGTCCGATGAG<br>GCGGGACGACACCAGGCGGTTCCCGTCCACCTGCCAGGCGAGCTGCGGTGCCATGA<br>TCTGATACACACGCACCTCGTAGAAATGTTGCCTCCTCATCATGAACTTGGACGTG<br>TATCTTTGCCTGACCTTGAAGAGGTGCAGTTCGGCATCCAGGACATTTTCCGTGGT<br>CGACACAGAGGACACGTTGAAGAAGAAGCTTCTGTGTAGTACATCTTTGTCGGGAA<br>AACTTCTGACGACGTTTGCGGAGAGCGGGTTGGGAAATCGCACGATCCCGTCCGGG<br>TCTGAAATGGTGTTGTACAGATCCAACATGTACTGTGGCGGCCTGAAGTGAATC
Brachyury2	EU685284
Dmbx	MF287224
Chordin	EU685285
Goosecoid	CGGCCCCGGCCTGGACCACCTCCCCGGTGCAGATGGCGATCGGCCAGGCGACCCAC<br>CCCGCCATGTTCGGGCGGCAGGGCCGGAGGAAGCGGCGGCACCGCACCATCTTCAC<br>CGAGGAGCAGCTGGAGCTGCTGGAGAAGACGTTCGAGAAGACGCACTACCCGGACG<br>TGCTGCTGCGGGAGGAGCTCGCCATGAAGGTGGAGCTGAAGGAGGAGAGAGTTGAG<br>GTATGGTTCAAGAACCGCCGTGCAAAGTGGCGCAAGCAGCAGAGGGAGGTGACCGA<br>GCGCACGACGAAGGCTGCCGACGACGCCTGCGACTCCGACATCGACGTCACGAGCA<br>TTGACGACGAGGATGAAGTCCGCAGCGTCTCGTCTGACGACGGAAAGGCCGTCTCT<br>CCTGTCTG
Lhx2/9a	MF287220
Netrin	HM359127
Nodal	EU685293

Development • Supplementary information

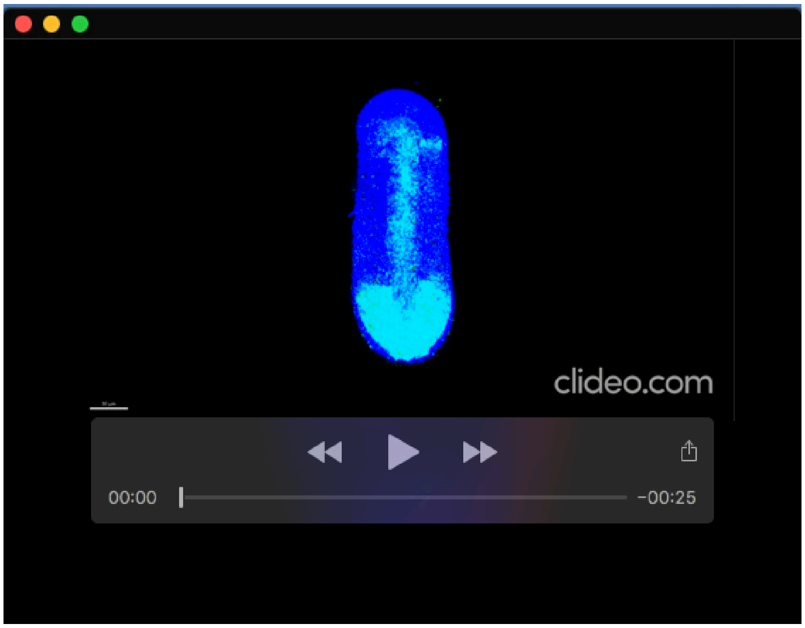

Movie 1. Movie presenting 3D views of one N2 stage control embryo after nuclei reconstruction.
