# Supplementary material for: Functions of the FGF signalling pathway in cephalochordates provide insight into the evolution of the prechordal plate
Source: Development. 2022 May 16;149(10):dev200252. doi: 10.1242/dev.200252 (PMC9188755; doi:10.1242/dev.200252)
Supplement: Reviewer comments [file develop-149-200252_review_history.pdf]

Functions of the FGF signalling pathway in cephalochordates provide insight into the evolution of the prechordal plate

Lydvina Meister, Hector Escriva and Stéphanie Bertrand
DOI: 10.1242/dev.200252

Editor: Cassandra Extavour

Review timeline
Original submission: 6 October 2021
Editorial decision: 18 January 2022
First revision received: 11 March 2022
Editorial decision: 29 March 2022
Second revision received: 31 March 2022
Accepted: 5 April 2022

Original submission

First decision letter

MS ID#: DEVELOP/2021/200252

MS TITLE: FGF signalling pathway function in cephalochordates enlightens the evolution of the prechordal plate

AUTHORS: Lydvina Meister, Hector Escriva, and Stephanie BERTRAND

I have now received all the referees' reports on the above manuscript, and have reached a decision. The referees' comments are appended below, or you can access them online: please go to BenchPressand click on the 'Manuscripts with Decisions' queue in the Author Area.

As you will see, the referees express considerable interest in your work, but have some significant criticisms and recommend a substantial revision of your manuscript before we can consider publication. I agree with the major points raised by these reviewers, and find that the two most critical areas for revision are (a) a more thorough and skeptical analysis of what the tissue fate actually is in the absence of FGF signaling, and (b) resisting the temptation to suggest that cephalochordates are 'primitive' or some kind of evolutionary precursor to living vertebrates. If you are able to revise the manuscript along the lines suggested, which may involve further experiments, I will be happy receive a revised version of the manuscript. Your revised paper will be re-reviewed by one or more of the original referees, and acceptance of your manuscript will depend on your addressing satisfactorily the reviewers' major concerns. Please also note that Development will normally permit only one round of major revision.

We are aware that you may be experiencing disruption to the normal running of your lab that make experimental revisions challenging. If it would be helpful, we encourage you to contact us to discuss your revision in greater detail. Please send us a point-by-point response indicating where you are able to address concerns raised (either experimentally or by changes to the text) and where you will not be able to do so within the normal timeframe of a revision. We will then provide further guidance. Please also note that we are happy to extend revision timeframes as necessary.

© 2022. Published by The Company of Biologists under the terms of the Creative Commons Attribution License (https://creativecommons.org/licenses/by/4.0/).

Please attend to all of the reviewers' comments and ensure that you clearly highlight all changes made in the revised manuscript. Please avoid using 'Tracked changes' in Word files as these are lost in PDF conversion. I should be grateful if you would also provide a point-by-point response detailing how you have dealt with the points raised by the reviewers in the 'Response to Reviewers' box. If you do not agree with any of their criticisms or suggestions please explain clearly why this is so.

Reviewer 1

Advance summary and potential significance to field

The authors present the novel idea that the vertebrate head mesoderm could have evolved by modulation of FGF signaling. If they can substantiate these claims, this paper would make an important addition to the literature. However, the present papers needs to rule out that this tissue is simply mis-specified after loss of FGF signaling.

Comments for the author

In this manuscript, the authors combine gene expression, lineage tracing and morphological analysis to study the anterior presumptive paraxial mesodermal cells and show that they acquire an anterior dorsal axial fate during gastrulation when FGF signal is inhibited, resulting in incorporation into the anterior notochord. They conclude that the amphioxus anterior notochord displays transient features of the prechordal plate. This work expands upon previous work by the authors on the general role of FGF signalling and somitogenesis from an evolutionary perspective (PNAS 2011) as well as a recent NatEcolEvo paper (2019) about the posterior mesoderm and the origin of the vertebrate head mesoderm . They present the novel idea that the vertebrate head mesoderm could have evolved by modulation of FGF signaling.

1. The main issue with the paper is that their data fall short of convincingly supporting their intriguing hypothesis. A counter-hypothesis is that inhibition of FGF-signaling may cause mis-specification of the tissue. The authors need to conclusively rule out the latter possibility
2. Fig.2 - The tissue looks strangely mis-specified; e.g. goosecoid is expressed everywhere in the treated animals. The claim that Hex is unaltered is not convincing. Nor is it clear that this domain does not overlap with nodal. The tissue appears to be expressing many combinations of genes. It is not helpful that their hypothetical diagrams do not reflect the patterns shown in individual images.
3. In Fig.3, how can the authors determine the tissue to which the different Kaede-labeled cells belong in the SU5402- inhibited animals? No structures are obvious. How representative are these images?
4. The authors should label individual images of their figures properly with A,B,C.. and refer to the specific images in the text with arrows, asterisks etc. Where are the transverse sections of Fig.2 positioned relative to whole animal?
5. For most experiments, the numbers of embryos are not indicated and no sections are provided. How representative are the images for each manipulation?

Reviewer 2

Advance summary and potential significance to field

The manuscript from Meister et al investigates the role of the FGF pathway during the anterior mesoderm development using experimental and descriptive approaches. They find that the presumptive paraxial mesoderm in Branchiostoma have a dorsal axial fate when the FGF signal is inhibited. The authors conclude further, that the anterior notochord shows characteristics of the prechordal plate. The authors interpret their findings in the context of head evolution in the vertebrates and suggest that changes in the FGF pathway played a role during the evolution of the complex vertebrate head.

The authors provide a manuscript that is very well structured, easy and interesting to read. I congratulate the authors for this. The conducted experiments are easy to follow and support their findings and conclusions.

© 2022. Published by The Company of Biologists under the terms of the Creative Commons Attribution License (https://creativecommons.org/licenses/by/4.0/).

The study is a valid contribution to the field and contributed one piece in the puzzle of the vertebrate head evolution. Regarding the experiments I have nothing to add or suggest. Given the current discussion about the vertebrate head evolution I think that the direction of interpretation the authors provide is also valid.

Comments for the author

I find the referencing very sparse. Albeit the introduction describes very well the state-of-the-art, one reference for previous findings (e.g. no FGF outside Metazoa) and discussions (e.g. whole topic of the Vertebrate head problem) is not giving sufficient credit to the previous research done in this field. E.g. also referencing only Delsuc et al for ascidians being sister to vertebrates is not considering the fact that in that paper amphioxus was sister to echinoderms. This does not provide the reader the correct information about the phylogenetic relationships, so here e.g. the corrections of the results from Delsuc et al need to be added.
So I ask for the benefit of the authors that they should provide more references in general - the manuscript is also gaining visibility by that. These are not the only points that need more references.
I see the tendency in the introduction to sell Branchiostoma as kind of a proto-vertebrate. This has been criticised before, and I suggest to tone down the sentences in the manuscript. E.g. "cephalochordates are considered vertebrate-like but with a simpler morphology and a prototypical chordate genome, and are thus good model systems to decipher how vertebrate novelties appeared”. At the end it is an animal like many others and evolution happened along all lineages. To me, this sentence is not necessary, so it can be deleted from the manuscript. "If we consider that the ancestral chordate was similar to amphioxus,…" is a similar statement that at the end does not mean anything with “similar”. Similarities need to be made more specific e.g. had a similar xx or yy.

<u>Reviewer 3</u>

Advance summary and potential significance to field

The prechordal plate is the most anterior part of axial mesoderm that is characterized by very specific expression of several genes such as Gsc and Six3 and plays essential roles in the development of vertebrate forebrain.
Originally, by examining the expression of Gsc gene in amphioxus embryos (2000), Neidert et al. concluded that amphioxus embryos lack a structure equivalent to vertebrate prechordal plate. In a recent study, however, Albuixech-Crespo and his colleagues (2017, Plos Biology) reached a different conclusion based on the different expression patterns of Hh, IrxB, FoxA2-1 and Six3/6 genes in amphioxus axial mesoderm and thought that the rostral notochordal tip of amphioxus embryos might be a possible prechordal plate homologue. Consistent with this, Andrews et al. (2021, Development) further showed that cell behavior within the rostral tip of amphioxus notochord is different from that within the middle and posterior regions. In this ms, Meister and his colleagues provided more evidence for this scenario. In addition, they also suggest that when inhibiting FGF signaling from the blastula stage, the first pair, but not the first three pairs of somites as they previously thought, turned their fates to notochord and later intercalated into the anterior notochord. Finally, based on their findings and those from previous studies, they proposed a refined model for the evolution of the vertebrate head. The ms is of certain novelties and would be interesting to readers working on the evolution of vertebrates. The conclusions in the ms are generally sound, but some of these need to be re-evaluated.

Comments for the author

Major comments:
1) the embryos treated with SU5402 are apparently wider but shorter than the control from the view of either morphology or gene expression of axial markers (like Chordin, Netrin and Admp and Gsc shown in Fig. 2). This might be caused by developmental delay, disruption of convergent extension, and/or cell fate change (from paraxial to axial mesoderm) by SU5402. All these possibilities should be considered to interpret their results, especially given that both Nodal and

© 2022. Published by The Company of Biologists under the terms of the Creative Commons Attribution License (https://creativecommons.org/licenses/by/4.0/).

Gsc expression patterns are highly dynamic during G6-N1 stage while the main finding in this part is heavily based on them.
2) it is really hard to say “FGF signalling pathway inhibition induces a change of fate of the anterior paraxial mesoderm territory corresponding to the presumptive first somite pair”, although the first somite pair show several different properties from the other ones as the authors listed in the Discussion section. First, double in situ experiment did not provide clear evidence for this. Indeed, after treatment, Gsc and Nodal were coexpressed in the most anterior mesoderm territory corresponding to the presumptive first somite pair at G6 stage, but their coexpression domain appear to extend to more posterior region at N1 stage at the level of the second somite pair. Second, cell-tracing experiment show no red signals in regions other than notochord all along the anterior two thirds of the treated embryos at T0 stage, which strongly suggested that not only the first but more anterior somite pairs were converted into notochord. Third, calculating ratio of cell numbers in the anterior notochord of the treated embryos to those in the anterior notochord plus somites of control embryos would provide certain clue to define how many somites were lost and intercalated in the notochord in the treated embryos, but it is a indirect way.
Examining Cer (at G6-N0 stage it is expressed only in the first somite pair) and Wnt8 expression (it is expressed in all somite pairs except the first one), or counting somite number of embryos at late neurula stage if possible, might be helpful.

Minor comments:
1) Line 104: it is better to provide a DIC picture and a brief description to show the morphological difference between the treated and control embryos at G6 N1, N3, T0 and L2 in this section.
1) Line 121: expression of ADMP gene and its similarity and difference with that of Gsc should be described.
2) Line 122: paraxial might be axial?
3) Line 344: to what stage the treatment was stopped should be described.
4) Discussion section is insightful but there is too many speculations.
5) Line 183-184: Albuixech-Crespo and his colleagues’ work (2017, Plos Biology) should be cited in this section.
6) Line 246: notochordregion > notochord region 7) Figure 2: the level of the sagittal section should be labeled in.
8) Figure 3: the scheme should be more precisely presented following the confocal images.
9) Amphioxus gene names should be used in a consistent way, either “Nodal” or “NODAL”.
10) Figure 5: authors from this group have proposed several versions of model to highlight the importance of modification of FGF function in the evolution of vertebrate head. It would be essential and interesting to examine if FGF inhibition has any effects on the development of amphioxus cerebral vesicle.

First revision

Author response to reviewers' comments

Please find below our answers to the reviewer comments. For clarity our answers are in blue.

Reviewer 1 Comments for the Author...
In this manuscript, the authors combine gene expression, lineage tracing and morphological analysis to study the anterior presumptive paraxial mesodermal cells and show that they acquire an anterior dorsal axial fate during gastrulation when FGF signal is inhibited, resulting in incorporation into the anterior notochord. They conclude that the amphioxus anterior notochord displays transient features of the prechordal plate. This work expands upon previous work by the authors on the general role of FGF signalling and somitogenesis from an evolutionary perspective (PNAS 2011) as well as a recent NatEcolEvo paper (2019) about the posterior mesoderm and the origin of the vertebrate head mesoderm . They present the novel idea that the vertebrate head mesoderm could have evolved by modulation of FGF signaling.

© 2022. Published by The Company of Biologists under the terms of the Creative Commons Attribution License (https://creativecommons.org/licenses/by/4.0/).

1. The main issue with the paper is that their data fall short of convincingly supporting their intriguing hypothesis. A counter-hypothesis is that inhibition of FGF-signaling may cause mis-specification of the tissue. The authors need to conclusively rule out the latter possibility

We are sorry we do not really understand this remark. We indeed show in amphioxus that the first anterior somite presumptive territory is mis-specified after FGF signalling pathway inhibition and expresses *Goosecoid* that is normally not expressed in this region of the embryo. We also show that the cells of this region, after FGF signalling pathway inhibition, incorporate into the anterior axial mesoderm. We hence don't understand what the counter-hypothesis means as it is here not an hypothesis: we showed using different techniques what is the fate of the anterior somite territory cells after FGF signalling inhibition in amphioxus. However, if the reviewer refers to our hypothesis concerning vertebrate evolution, we only propose, based on the data obtained in this paper, and on other data obtained by our group (Bertrand et al., 2011, PNAS and Aldea et al., 2019, Nature Ecology and Evolution), as cited by the reviewer, that changes of the FGF signalling pathway developmental roles during evolution might have participated to the modification of the anterior mesoderm in the vertebrate lineage.

2. Fig.2 - The tissue looks strangely mis-specified; e.g. goosecoid is expressed everywhere in the treated animals. The claim that Hex is unaltered is not convincing. Nor is it clear that this domain does not overlap with nodal. The tissue appears to be expressing many combinations of genes. It is not helpful that their hypothetical diagrams do not reflect the patterns shown in individual images.

We are sorry we don't understand to which tissue the reviewer refers to. *Goosecoid* is not expressed "everywhere": it is expressed in both control and treated embryos in the axial mesoderm and neural plate, and in treated embryos an additional domain is observed in the paraxial anterior region that also expresses *Nodal*. We have explained in more details the expression patterns of the different genes used in the study and now we also provide new images. We have deleted the diagrams and separated the Figure 2 in two different figures so that we hope the pictures are now clearer for the reader. We have also modified the colors used for the fluorescent pictures in order to facilitate the reading by color-blind people.

3. In Fig.3, how can the authors determine the tissue to which the different Kaede- labeled cells belong in the SU5402- inhibited animals? No structures are obvious. How representative are these images?

As for the control embryos, the labelled structures are clear to determine, but we understand that for non-specialists the images may not be clear enough. We have modified Fig. 3 (now Fig. 4) so that we hope it is now easier to visualize. Concerning the representativity of the images, as written in the result section, "In the control embryos that showed red fluorescence in the ventral epidermis, as expected, the somites were red-labelled all along the body axis (Figure 3, n=4/4). On the other hand, in the correctly photoconverted treated embryos, while the posterior somites were red, the notochord was labelled in the anterior part of the embryo, in the region where the somites are absent (Figure 3, n=7/7)."

4. The authors should label individual images of their figures properly with A,B,C.. and refer to the specific images in the text with arrows, asterisks etc. Where are the transverse sections of Fig.2 positioned relative to whole animal?

For the confocal images of Fig. 2, in order to avoid overloading the images, we provided supplementary figures to indicate the position of the section by presenting raw imageJ orthogonal views images. However, as both Reviewer 1 and Reviewer 3 asked for it, we have modified Figure 2 accordingly and separated the data in two figures. We have also labelled the panels according to the reviewer recommendation and added arrowheads when necessary.

5. For most experiments, the numbers of embryos are not indicated and no sections are provided. How representative are the images for each manipulation?

We don't understand this comment. We indicated the number of embryos used for each experiment and explained if the images are representative in the version read by the reviewer:

© 2022. Published by The Company of Biologists under the terms of the Creative Commons Attribution License (https://creativecommons.org/licenses/by/4.0/).

Legend of Figure 1: “At least ten embryos per stage were analyzed and they all showed the same pattern.”

Legend of Figure 2: “At least five embryos per stage were analyzed and they all showed the same pattern”

Concerning Figure 3 in the results: “In the control embryos that showed red fluorescence in the ventral epidermis, as expected, the somites were red-labelled all along the body axis (Figure 3, n=4/4). On the other hand, in the correctly photoconverted treated embryos, while the posterior somites were red, the notochord was labelled in the anterior part of the embryo, in the region where the somites are absent (Figure 3, n=7/7).”

Legend of Figure 4: “One-way ANOVA and post-hoc Tukey test analysis results are shown: N.S. = non-significant, ** = p<0,01, *** = p<0,001, **** = p<0,0001, n=4 embryos.”

Reviewer 2 Comments for the Author...
I find the referencing very sparse. Albeit the introduction describes very well the state- of-the-art, one reference for previous findings (e.g. no FGF outside Metazoa) and discussions (e.g. whole topic of the Vertebrate head problem) is not giving sufficient credit to the previous research done in this field. E.g. also referencing only Delsuc et al for ascidians being sister to vertebrates is not considering the fact that in that paper amphioxus was sister to echinoderms. This does not provide the reader the correct information about the phylogenetic relationships, so here e.g. the corrections of the results from Delsuc et al need to be added. So I ask for the benefit of the authors that they should provide more references in general - the manuscript is also gaining visibility by that. These are not the only points that need more references.

We would like to thank the reviewer for this comment that helped us improve the manuscript. We have added 44 references to give credit to all previous research and the text is now citing 78 articles.

I see the tendency in the introduction to sell Branchiostoma as kind of a proto- vertebrate. This has been criticised before, and I suggest to tone down the sentences in the manuscript. E.g. "cephalochordates are considered vertebrate-like but with a simpler morphology and a prototypical chordate genome, and are thus good model systems to decipher how vertebrate novelties appeared”. At the end it is an animal like many others and evolution happened along all lineages. To me, this sentence is not necessary, so it can be deleted from the manuscript.

We completely agree with the concern of the reviewer and did not want to seem to consider cephalochordates as something which does not exist: a living fossil. We have modified the sentence so that it is now as follows: “On the other hand, cephalochordates have retained chordate characters shared with vertebrates such as the presence of a dorsal organizer (Garcia-Fernandez, D'Aniello et al. 2007, Yu, Satou et al. 2007, Bertrand and Escriva 2011, Le Petillon, Luxardi et al. 2017) and have conserved at least one orthologue of almost all the genes that were predicted in the putative chordate ancestor”.

"If we consider that the ancestral chordate was similar to amphioxus,…" is a similar statement that at the end does not mean anything with “similar”. Similarities need to be made more specific e.g. had a similar xx or yy.

Thank you for this remark. We have changed the sentence to be more precise, and now it reads as follows: “If we consider that the ancestral chordate had the same mesodermal compartments as observed in amphioxus, except that it had a permanent prechordal plate, we can propose a new evolutionary framework for the appearance of the complex head of vertebrates”

Reviewer 3 Comments for the Author...
Major comments:
1) the embryos treated with SU5402 are apparently wider but shorter than the control from the view of either morphology or gene expression of axial markers (like Chordin, Netrin and Admp and Gsc shown in Fig. 2). This might be caused by developmental delay, disruption of convergent

© 2022. Published by The Company of Biologists under the terms of the Creative Commons Attribution License (https://creativecommons.org/licenses/by/4.0/).

extension, and/or cell fate change (from paraxial to axial mesoderm) by SU5402. All these possibilities should be considered to interpret their results, especially given that both Nodal and Gsc expression patterns are highly dynamic during G6-N1 stage while the main finding in this part is heavily based on them.

We have added DIC images of control and SU5402-treated embryos in Supplementary Figure 1 (as also proposed in the minor comment point 1). We are now discussing this point in the corresponding section. We indeed choose to look at the expression pattern of several genes at the G6 and N1 stage in order to cover the dynamics of expression of the different genes.

2) it is really hard to say “FGF signalling pathway inhibition induces a change of fate of the anterior paraxial mesoderm territory corresponding to the presumptive first somite pair”, although the first somite pair show several different properties from the other ones as the authors listed in the Discussion section.
-First, double in situ experiment did not provide clear evidence for this. Indeed, after treatment, Gsc and Nodal were coexpressed in the most anterior mesoderm territory corresponding to the presumptive first somite pair at G6 stage, but their coexpression domain appear to extend to more posterior region at N1 stage at the level of the second somite pair.

We have added in this version of the manuscript *in situ* hybridizations for *Dmbx*, which is expressed in the anterior axial mesoderm as well as in the first somite pair/presumptive first somite pair at G6 and N1 in control embryos. In SU5402-treated embryos, we observed that the region of *Nodal* anterior paraxial expression that overlap with the *Goosecoid* expression domain corresponds to the same region as the one for which *Nodal*/*Dmbx* co-expression is observed. We have also added orthogonal views images at the level of the presumptive second somite pair for *Nodal*/*Goosecoid in situ* hybridization images showing that there is no expression overlap in the paraxial region at this level.

-Second, cell-tracing experiment show no red signals in regions other than notochord all along the anterior two thirds of the treated embryos at T0 stage, which strongly suggested that not only the first but more anterior somite pairs were converted into notochord.

We understand the remark of the reviewer. The kaede photoconverted embryos were imaged in vivo and are not totally oriented lateral which gives this impression. We hope that the new figure makes it clearer.

-Third, calculating ratio of cell numbers in the anterior notochord of the treated embryos to those in the anterior notochord plus somites of control embryos would provide certain clue to define how many somites were lost and intercalated in the notochord in the treated embryos, but it is a indirect way. Examining Cer (at G6-N0 stage it is expressed only in the first somite pair) and Wnt8 expression (it is expressed in all somite pairs except the first one), or counting somite number of embryos at late neurula stage if possible, might be helpful.

We understand the concern of the reviewer. Since our first experiments on the role of the FGF signalling pathway during amphioxus embryogenesis, we have looked for a way to define precisely how many somites are lost upon this signal inhibition. However, there is no direct way. Indeed, by looking for example at the expression of *Cerberus* (which is expressed in the first somite pair), if the expression is maintained it will give us no information, and if the expression is lost, it will mean that the first somite pair is lost but we will not be able to define if the second or the third pair for example are also lost. The same holds for *Wnt8*. If all the somites that form after the treatment express *Wnt8*, it would mean that the first pair is lost, but we will not be able to define if more pairs are lost. However, we have looked at the expression of *Dmbx*, which is expressed in the anterior axial mesoderm as well as in the first somite pair/presumptive first somite pair at G6 and N1 in control embryos. We show that in SU5402 treated embryos, the expression is also observed at G6 in the anterior axial mesoderm as well as in the anterior paraxial domain expressing *Nodal*. At N1, the paraxial domain of *Dmbx* expression overlapping with *Nodal* labelling corresponds to the paraxial anterior region in which we observe *Goosecoid* expression, supporting the fact that only the region of the first presumptive somite pair is affected by the treatment. We have added these data in the manuscript (see Figure 3). We have

© 2022. Published by The Company of Biologists under the terms of the Creative Commons Attribution License (https://creativecommons.org/licenses/by/4.0/).

also counted the somites at the T0 stages and added the corresponding figure as Supplementary Figure 7.

Minor comments:
1) Line 104: it is better to provide a DIC picture and a brief description to show the morphological difference between the treated and control embryos at G6, N1, N3, T0 and L2 in this section.

We have added in Supplementary Figure 1 DIC pictures of control and treated embryos.

1) Line 121: expression of ADMP gene and its similarity and difference with that of Gsc should be described.

We would like to thank the reviewer for this remark. We detail now more precisely the expression pattern of *Netrin*, *Chordin*, *ADMP* and *Gossecoid* and we have added a supplementary figure with the full orthogonal views in order for the reader to better visualize the dynamic expression of these genes (see Supplementary Figure 2).

2) Line 122: paraxial might be axial?

The reviewer is perfectly right, we modified the text accordingly

3) Line 344: to what stage the treatment was stopped should be described.

We have added the information in the method section.

4) Discussion section is insightful but there is too many speculations.

The two other reviewers did not make such a comment. We believe that the interest of using a model system such as amphioxus is to give the opportunity to propose new hypothesis. We also consider we are not overstating our results but that we are discussing, in light with the litterature, some hypothesis about mesoderm evolution in chordates that could be useful for the community. We have modified the discussion so that we hope it is now less speculative.

5) Line 183-184: Albuixech-Crespo and his colleagues' work (2017, Plos Biology) should be cited in this section.

We have added this reference.

6) Line 246: notochordregion > notochord region Thank you, we corrected the typo.

7) Figure 2: the level of the sagittal section should be labeled in.

We have modified all the figures presenting confocal images so that the position of the sections are now indicated.

8) Figure 3: the scheme should be more precisely presented following the confocal images.

We have deleted the scheme and now show more detailed images from the confocal data. We hope it will be now clearer for the reader.

9) Amphioxus gene names should be used in a consistent way, either "Nodal" or "NODAL".

We are using Nodal all through the manuscript.

10) Figure 5: authors from this group have proposed several versions of model to highlight the importance of modification of FGF function in the evolution of vertebrate head. It would be essential and interesting to examine if FGF inhibition has any effects on the development of amphioxus cerebral vesicle.

© 2022. Published by The Company of Biologists under the terms of the Creative Commons Attribution License (https://creativecommons.org/licenses/by/4.0/).

We understand the remark of the reviewer, and the question of the effects on the cerebral vesicle is an important issue that requires many additional experiments. This is why we believe that it is out of the scope of this manuscript.

Second decision letter

MS ID#: DEVELOP/2021/200252

MS TITLE: FGF signalling pathway function in cephalochordates enlightens the evolution of the prechordal plate

AUTHORS: Lydvina Meister, Hector Escriva, and Stephanie BERTRAND

I have now received all the referees reports on the above manuscript, and have reached a decision. The referees' comments are appended below, or you can access them online: please go to BenchPress and click on the 'Manuscripts with Decisions' queue in the Author Area.

The overall evaluation is positive and we would like to publish a revised manuscript in Development, provided that the referees' comments can be satisfactorily addressed. Please attend to all of the reviewers' comments in your revised manuscript and detail them in your point-by-point response. If you do not agree with any of their criticisms or suggestions explain clearly why this is so. If it would be helpful, you are welcome to contact us to discuss your revision in greater detail. Please send us a point-by-point response indicating your plans for addressing the referee's comments, and we will look over this and provide further guidance. No new experiments are needed for this revision. However, I do find that the comments of Reviewer 1 regarding acknowledging potential alternative interpretations of a number of your findings, are fair comments. I would therefore invite you to consider including discussion of these potential interpretations, and how they might suggest alternative explanatory hypotheses, in your revised manuscript. I will not send the revised manuscript out for another round of review, but instead will review it myself, to try to provide a faster route to acceptance. Thank you for considering these revisions, which I believe will not detract from your paper at all, but rather strengthen it.

Reviewer 1

Advance summary and potential significance to field

The authors combine gene expression, lineage tracing and morphological analysis to study the anterior presumptive paraxial mesodermal cells. The results show that they acquire an anterior dorsal axial fate during gastrulation when FGF signal is inhibited, resulting in incorporation into the anterior notochord. They present the novel idea that the vertebrate head mesoderm could have evolved by modulation of FGF signaling.

Comments for the author

In this revised manuscript, the authors have made several improvements. However, the authors has not adequately dealt with the first two comments in my original review:
1. The main issue with the paper is that their data fall short of convincingly supporting their intriguing hypothesis. A counter-hypothesis is that inhibition of FGF-signaling may cause mis-specification of the tissue. The authors need to conclusively rule out the latter possibility.
The authors should consider alternative hypotheses and these should be reflected in Fig. 6. For example, it is possible that the ancestor did not have a prechordal plate. They should discuss the situation in other chordates and consider other possible scenarios in their summary scheme.
2. Fig.2 - The tissue looks strangely mis-specified; e.g. goosecoid is expressed everywhere in the treated animals. The claim that Hex is unaltered is not convincing. Nor is it clear that this domain does not overlap with nodal. The tissue appears to be expressing many combinations of

© 2022. Published by The Company of Biologists under the terms of the Creative Commons Attribution License (https://creativecommons.org/licenses/by/4.0/).

genes. It is not helpful that their hypothetical diagrams do not reflect the patterns shown in individual images.
The authors' statement that Hex expression is comparable between control and treated embryos (134-136) is still not supported by the images in Figure 2. The suggestion that Goosecoid expression is similar between control and treated embryos (162-166) is also difficult to conclude from the images shown in Figure 3. This is particularly important since it is possible that the SU5402-treated embryos are developmentally delayed which the authors should have considered as this complicates the analysis. Comparing ratios of nuclei as described for Figure 5 may not reliably reveal the number of somite pairs lost in SU5402-treated embryos.
This may be further complicated by the structure/morphology of treated embryos, which are quite different from controls, and that there may be developmental delays.

Minor points:
Please state the sample size in the legend of Figure 4. It would be good to indicate color coding in all figures (e.g. 1, 4, 5)
Some text descriptions of the expression patterns shown in Figure 2 should be clarified (e.g. lines 147-150).
The use of the word "enlightens" sounds strange, is unclear and makes interpretation of the title difficult.

Reviewer 2

Advance summary and potential significance to field

The revised version of the manuscript has addressed my points raised. This manuscript is an important contribution to understand the potential role of the FGF pathway for head mesoderm evolution.

Comments for the author

My points have been addressed.

Reviewer 3

Advance summary and potential significance to field

The work sheds new insights into the evolution of prechordate and shows how changes of FGF functions have contributed to it during chordate evolution.

Comments for the author

The authors have well dealt with all my comments and concerns and I have no further comments.

Second revision

Author response to reviewers' comments

For clarity our answers to the first round of reviews are in *blue and italics* and our answers to the second round of reviews in green.

Reviewer 1 Comments for the Author...
In this revised manuscript, the authors have made several improvements. However, the authors has not adequately dealt with the first two comments in my original review:

© 2022. Published by The Company of Biologists under the terms of the Creative Commons Attribution License (https://creativecommons.org/licenses/by/4.0/).

1. The main issue with the paper is that their data fall short of convincingly supporting their intriguing hypothesis. A counter-hypothesis is that inhibition of FGF- signaling may cause mis-specification of the tissue. The authors need to conclusively rule out the latter possibility.

> *Our previous answer in R1: "We are sorry we do not really understand this remark. We indeed show in amphioxus that the first anterior somite presumptive territory is mis-specified after FGF signalling pathway inhibition and expresses Goosecoid that is normally not expressed in this region of the embryo. We also show that the cells of this region, after FGF signalling pathway inhibition, incorporate into the anterior axial mesoderm. We hence don't understand what the counter-hypothesis means as it is here not an hypothesis: we showed using different techniques what is the fate of the anterior somite territory cells after FGF signalling inhibition in amphioxus. However, if the reviewer refers to our hypothesis concerning vertebrate evolution, we only propose, based on the data obtained in this paper, and on other data obtained by our group (Bertrand et al., 2011, PNAS and Aldea et al., 2019, Nature Ecology and Evolution), as cited by the reviewer, that changes of the FGF signalling pathway developmental roles during evolution might have participated to the modification of the anterior mesoderm in the vertebrate lineage."*

The authors should consider alternative hypotheses and these should be reflected in Fig. 6. For example, it is possible that the ancestor did not have a prechordal plate. They should discuss the situation in other chordates and consider other possible scenarios in their summary scheme.

We now better understand the meaning of the reviewer remark. In this revised version we decided not to propose alternative scenario on the evolution of the paraxial mesoderm that we previously proposed because there were based on previous studies, but to provide an alternative scenario in which we considered another hypothesis concerning the ancestral state and evolution of the the axial mesoderm in chordates. We are discussing this alternative hypothesis in the main text and have added the corresponding schematic in Figure 6 panel B. The last section of the Discussion part of the manuscript now reads as follows (we indicated the modificatons in red):

"Based on our previous work we formulated a multistep hypothetical evolutionary scenario underlying vertebrate head mesoderm origin in which the chordate ancestor had a paraxial mesoderm segmented all along its antero-posterior axis as observed in extant cephalochordates (Bertrand, Camasses et al. 2011, Aldea, Subirana et al. 2019).
Segregation of the lateral/ventral mesoderm from the paraxial mesoderm, associated to a loss of segmentation of the lateral/ventral mesoderm, was the first step (Aldea, Subirana et al. 2019). The second step was the regionalization of the lateral/ventral mesoderm. Finally, in a third step, the loss of the anterior paraxial mesoderm, probably through a modification of the role of the FGF signalling pathway, would have reduced the developmental constraints and allowed the lateral plate mesoderm to "colonize" this anterior dorsal territory for the formation of a "novel" muscular system. The present study allows us to refine this hypothesis, by including a scenario for the evolution of the prechordal plate. Regarding the axial mesoderm, although it is accepted that the ancestral chordate possessed a notochord, which is synapomorphic for this group (Annona, Holland et al. 2015), it is still unclear whether it ran along its entire antero-posterior axis. Indeed, each group of extant chordates shows a different pattern. In cephalochordates, the notochord extands from the anterior of the cerebral vesicle to the posterior end of the animal. In tunicates, the notochord is present only in the tail, whereas in vertebrates the anterior axial mesoderm forms the prechordal plate anterior to the otic vesicle. The fossil record can provide some support although only larval or adult stages are available, and the interpretation in terms of structures observed and phylogenetic position of the species described is subject to much debate (Holland and Chen 2001, Swalla and Smith 2008). Hence, in the two fossil species that can be recognized as potential early Cambrian chordates, *Pikaia gracilens* and *Yunnanozoon lividum* (Chen, Dzik et al. 1995, Morris and Caron 2012), there is no notochord in the anterior region. This suggests that the ancestral chordate had a prechordal plate-like structure in the anterior region, or that it had neither anterior notochord nor prechordal plate. If we consider that the ancestral chordate had a prechordal plate-like anterior axial mesoderm, which is the most parsimonious hypothesis if we consider that cephalochordates possess a transient prechordal plate as we and others suggest (Albuixech-Crespo, Lopez-Blanch et al. 2017, Andrews, Pönisch et al. 2021, Ferran, Irimia et al. 2022), we can propose a new evolutionary

© 2022. Published by The Company of Biologists under the terms of the Creative Commons Attribution License (https://creativecommons.org/licenses/by/4.0/).

framework for the appearance of the complex vertebrate head (Fig. 6 A). Thus, during vertebrate evolution, the role of the FGF signal changed, resulting in the acquisition of new functions and the loss of its contribution to the anterior mesoderm formation, with similar effects to those we observed in amphioxus after FGFR inhibition. This would have led to the formation of a bigger prechordal plate not flanked by somites but by the anterior lateral plate mesoderm. The latter would have lost the constraints imposed by the neighboring somites and gained the possibility to form new tissues, among which the muscles derived from the cranial/pharyngeal mesoderm. In this scenario, the enlargement of the prechordal plate territory, and the inclusion of the most anterior paraxial mesoderm in this structure, could explain the larger anterior brain observed in vertebrates *versus* non-vertebrate chordates, and the capacity of the prechordal plate to also form some head muscles. Indeed, the anterior brain of vertebrates is induced by the prechordal plate, which is sometimes described as the "head organizer" in non-amniote vertebrates (De Robertis, Fainsod et al. 1994), and the prechordal plate gives rise to the extraocular muscles in vertebrates (Noden and Francis-West 2006). In this scenario, cephalochordates would also have derived from the ancestral state, with the acquisition of a notochord growing anteriorly. This cephalochordate synapomorphy would have resulted from the duplication of the *Brachyury* gene and gain of expression of one of the duplicate in the anterior axial mesoderm. However, if we consider that the chordate ancestor did not have a prechordal plate-like structure or a notochord, we can draw another hypothetical scenario (Fig. 6 B). Thus, cephalochordates would have acquired an anterior notochord while during vertebrate evolution, the loss of the anterior segmented paraxial mesoderm and associated developmental constraints would have led to the remodeling of the axial and lateral anterior mesoderm territories, resulting in the appearance of the prechordal plate and of the cranial/pharyngeal mesoderm.

To conclude, by studying the fate of the anterior paraxial mesoderm after FGF signalling pathway inhibition in cephalochordates, we highlighted the peculiarities of the anterior notochord in this chordate lineage and bring new insights into how the complex head of vertebrates, with a large brain and specific muscles, might have emerged during evolution."

Figure 6B is now as follows:

© 2022. Published by The Company of Biologists under the terms of the Creative Commons Attribution License (https://creativecommons.org/licenses/by/4.0/).

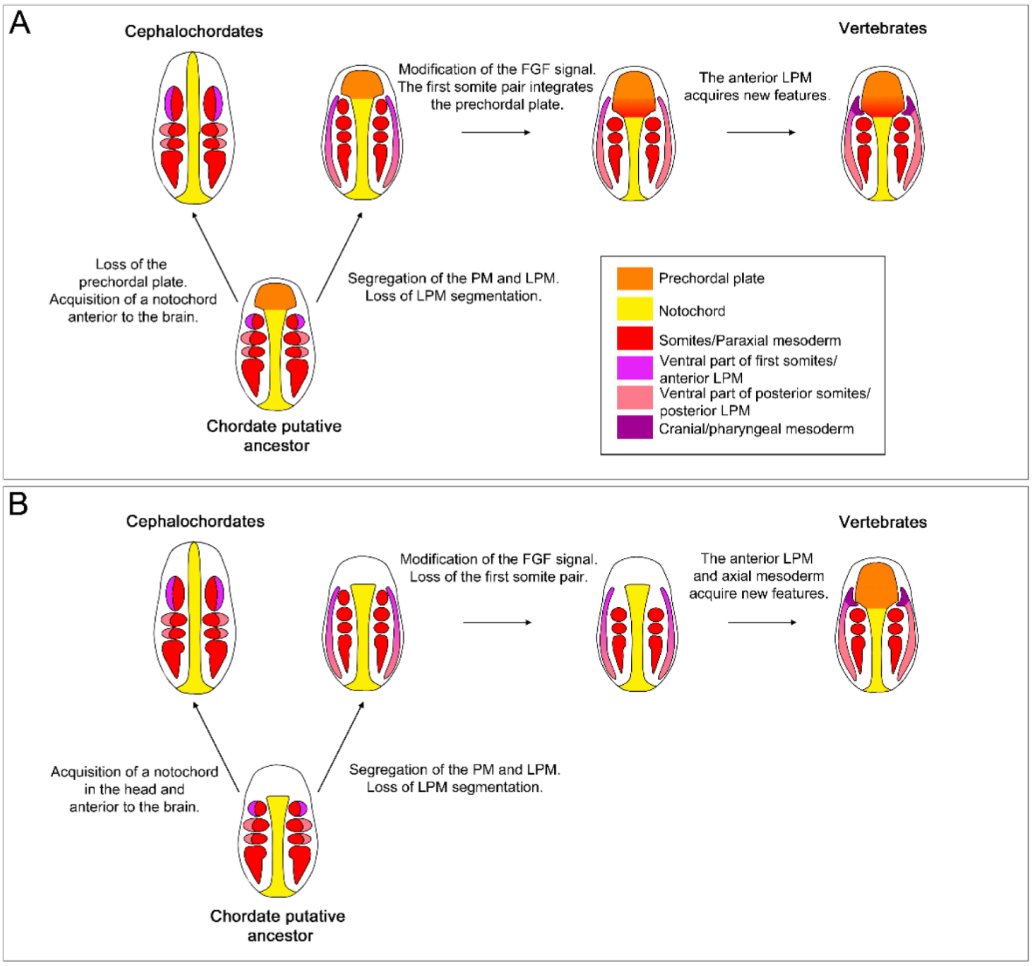

Figure 6. Two scenarios for the evolution of the anterior mesoderm in chordates. Schematics of embryos at the early somitogenesis stage are presented as dorsal views with anterior to the top. Arrows correspond to evolutionary steps. Color coding of different mesodermal territories is shown in the lower right of panel (A). PM, paraxial mesoderm; LPM, lateral plate mesoderm. (A) In this scenario, we considered that the chordate ancestor had a prechordal plate- like structure as observed transiently in amphioxus. (B) In this scenario, we considered that the chordate ancestor had neither an anterior notochord nor a prechordal plate.

2. Fig.2 - The tissue looks strangely mis-specified; e.g. goosecoid is expressed everywhere in the treated animals. The claim that Hex is unaltered is not convincing. Nor is it clear that this domain does not overlap with nodal. The tissue appears to be expressing many combinations of genes. It is not helpful that their hypothetical diagrams do not reflect the patterns shown in individual images.

> *Our previous answer in R1: "We are sorry we don't understand to which tissue the reviewer refers to. Goosecoid is not expressed "everywhere": it is expressed in both control and treated embryos in the axial mesoderm and neural plate, and in treated embryos an additional domain is observed in the paraxial anterior region that also expresses Nodal. We have explained in more details the expression patterns of the different genes used in the study and now we also provide new images. We have deleted the diagrams and separated the Figure 2 in two different figures so that we hope the pictures are now clearer for the reader. We have also modified the colors used for the fluorescent pictures in order to facilitate the reading by color-blind people."*

The authors' statement that Hex expression is comparable between control and treated embryos (134-136) is still not supported by the images in Figure 2. The suggestion that Goosecoid expression is similar between control and treated embryos (162-166) is also difficult to conclude

© 2022. Published by The Company of Biologists under the terms of the Creative Commons Attribution License (https://creativecommons.org/licenses/by/4.0/).

from the images shown in Figure 3. This is particularly important since it is possible that the SU5402-treated embryos are developmentally delayed which the authors should have considered as this complicates the analysis. Comparing ratios of nuclei as described for Figure 5 may not reliably reveal the number of somite pairs lost in SU5402-treated embryos. This may be further complicated by the structure/morphology of treated embryos, which are quite different from controls, and that there may be developmental delays.

We understand the concern of the reviewer. There is indeed a little developmental delay at early stages in SU5402-treated embryos, which we already mentionned in our previous version (R1):

- line 127: “We observed that the treated embryos were a little bit **delayed** at 15 hpf, and were hence **less elongated than controls** (Supp. Fig. 1). However, at 18 hpf, although no somites were detected, the SU5402-treated embryos showed an overall size and morphology similar to controls when oberved under the light microscope (Supp. Fig. 1)”
- line 141: “At this stage, Chordin was expressed in the neural plate of control embryos (Fig. 2 S-T, Supp. Fig. 2 C) whereas in treated embryos both neural plate and underlying axial mesendoderm were labelled (Fig. 2 W-X, Supp. Fig. 2 D), **probably due to a little developmental delay** compared to controls at this stage.”
- line 160: “At 18 hpf, Goosecoid expression was similar to controls but was still observed in the dorsal axial mesendoderm in the region posterior to the presumptive cerebral vesicle, again probably due to a little **developmental delay**”

To better integrate this point in the expression pattern comparison, we have modified the descriptons of *Hex* and *Goosecoid* expression that now reads as follows:

- line 131: “In treated embryos, **although they show a little delay and a flatter morphology**, the expression of the endodermal gene *Hex* (Yu, Satou et al. 2007) was similar to control embryos and no overlapping with Nodal expression was detected in either control or treated embryos at 15 hpf or 18 hpf (Fig. 2 A-H). **Indeed, *Hex* is expressed in both cases in the presumptive endoderm until the most anterior region, adjacent to the anterior axial Nodal expression territory** (Supp. Fig. 2 A-D).”

- line 161: “In SU5402-treated embryos, the expression was observed at 15hpf in the whole axial dorsal mesendoderm and in the presumptive neural plate, **which are wider than in controls due to the developmental delay**. Compared to control embryos, an additional small anterior paraxial domain of expression, overlapping with *Nodal* expression, was detected (Fig. 3 **I**-P, Supp. Fig. 2 N). At 18 hpf, *Goosecoid* expression was observed in the same axial regions as in controls **but was still detected in the dorsal axial mesendoderm in the region posterior to the presumptive cerebral vesicle, again probably due to a little developmental delay** (Fig. 3 A’-J’, Supp. Fig. 2 P).”

We have also added the full orthogonal views for *Hex*/*Nodal* double *in situ* hybridization in the Supplementary Figure 2 to further support the description.

Concerning the nuclei counting, to indegrate the fact that the delay/morphology might bias our comparison between control and treated embryos, we have modified the end of the paragraph that now reads as follows:

“These data suggest that the presumptive paraxial region that integrates the notochord in SU5402-treated embryos corresponds to the presumptive first somite pair territory. **However, as SU5402-treated embryos show a different morphology than controls and a developmental delay at early stages (Supp. Fig. 1), we cannot completely rule out the possibility that more somite pairs are lost**. On the other hand, the loss of only the first pair after FGF signalling pathway inhibition is consistent with the *in situ* hybridization data, and with somite numbers at later T0 (36hpf) stage (Supp. Fig. 7).”

© 2022. Published by The Company of Biologists under the terms of the Creative Commons Attribution License (https://creativecommons.org/licenses/by/4.0/).

Minor points:

Please state the sample size in the legend of Figure 4. It would be good to indicate color coding in all figures (e.g. 1, 4, 5)

We have added the following sentence in Figure 4 legend: “One representative embryo is presented for control (n=4/4) and SU5402-treated conditions (n=7/7).”.
We have modified the three figures (1, 4, 5) so that the color code is now indicated both in the legend and in the figure itself.

Some text descriptions of the expression patterns shown in Figure 2 should be clarified (e.g. lines 147-150).

We have modified the sentence that now reads as follows:
line 150: “ADMP was also expressed at the two studied stages in the neural plate region all along the antero-posterior axis of control and SU5402-treated embryos, and no overlap with the paraxial domain region expressing Nodal was observed”

The use of the word “enlightens” sounds strange, is unclear and makes interpretation of the title difficult.

We have modified the title that is now: “FGF signalling pathway function in cephalochordates provides insight into the evolution of the prechordal plate”

Third decision letter

MS ID#: DEVELOP/2021/200252

MS TITLE: FGF signalling pathway function in cephalochordates provides insight into the evolution of the prechordal plate

AUTHORS: Lydvina Meister, Hector Escriva, and Stephanie BERTRAND
ARTICLE TYPE: Research Article

I am happy to tell you that your manuscript has been accepted for publication in Development, pending our standard ethics checks.

© 2022. Published by The Company of Biologists under the terms of the Creative Commons Attribution License (https://creativecommons.org/licenses/by/4.0/).
